# Effects of mobile-supervised question-driven collaborative dialogues on EFL learners’ communication strategy use and academic oral English performance

**DOI:** 10.3389/fpsyg.2023.1142651

**Published:** 2023-07-03

**Authors:** Yuesheng Cai, Lawrence Jun Zhang

**Affiliations:** ^1^School of Foreign Languages, Shenzhen Technology University, Shenzhen, China; ^2^Faculty of Education and Social Work, The University of Auckland, Auckland, New Zealand

**Keywords:** mobile-supervised collaborative dialogues, academic L2 oral performance, fieldspecific oral data elicit questions, mean length of run, speaker compensatory communication strategies

## Abstract

This study investigated the effects of mobile-supervised question-driven collaborative dialogues (QDCDs) on reducing lower-intermediate-level English as a foreign language (EFL) participants’ tendency of their first language (L1) use in academic collaborative dialogues and on improving their academic foreign language (L2) oral performance. Throughout a whole semester, one group (*n* = 20) was involved in a mobile-supervised QDCDs intervention and a control group (*n* = 26) was involved in QDCDs with no supervision. Three semi-open-ended and three closed-ended academic questions were used to elicit pre-and post-study oral performance data from the participants. Independent-samples *t*-tests showed that after the intervention, the mobile-supervised group outperformed its control counterpart in a statistically significant manner in terms of Non-repeated L2 word production (NRW), T-unit count (TC), and Mean Length of Run after pruning (MLRP). The intervention group also significantly reduced their dependence on their L1-based speaker compensatory communication strategies (SC-CSs) in QDCDs. These results suggest that the intervention group outperformed the control group in their L2 academic oral performance and their language use tendency moves toward the L2 during QDCD. Based on the findings, we conclude that, even though L1 oral output may temporally enhance the quality of lower-intermediate-level EFL learners’ tasks, it may inhibit their academic oral proficiency development in the long run. Methods for fragmental bilingual oral output analysis are introduced. Pedagogical implications of the findings for MALL are also discussed.

## Introduction

Kowal and Swain (1997) observed that EFL learners’ receptive skills such as listening and reading can reach native-speaker levels; however, their productive skills, speaking and writing, rarely do so. In academic contexts, where analytical thinking and logical analysis are naturally expected, EFL learners face more challenges than in daily conversation contexts (Ong and Zhang, 2010; Zhang and Zhang, 2020). Towell et al. (1996) suggested that learners’ use of the target language is helpful in transforming the declarative knowledge stored in their long-term memory into procedural knowledge, and thus, in improving their oral proficiency (see also Perkins and Zhang, 2022). According to this theory, declarative knowledge requires more attention of the speaker than procedural knowledge, so less of it can be processed at one time than procedural knowledge. Therefore, when spontaneity is required in authentic oral communication, the declarative knowledge is not enough to support the output. Influenced by Krashen’s (1981) Acquisition-Learning Hypothesis, many researchers posited that teaching entirely through an L2 may help to develop learners’ oral performance (Macaro, 2001). Nevertheless, since the turn of the century, an increasing number of positive reports on L1 use in L2 classroom instructions have been published (Behan et al., 1997; Swain and Lapkin, 2000). These reports usually were based on the evaluation of EFL learners’ immediate task performance rather than on their long-term oral performance development. For long-term oral development, we posit that, if the L2 learners have grasped a lot of L2 knowledge but it is stored as declarative knowledge, which is slow in retrieving in oral communication, it is necessary to promote dominant L2 communication to realize the transformation of the declarative knowledge into procedural knowledge. The current study was designed to address the assumption.

In EFL contexts, where authentic communicative use of English is minimal, communicative pedagogical approaches have been found to be able to improve EFL learners’ oral performance (Li et al., 2020; Yang et al., 2020; Zhang and Zhang, 2020, 2021; Li and Zhang, 2021), as they provide learners with opportunities for using their L2. However, in real practice, the potential of L2 use during such communicative approaches has not been brought into its full capacity, because the evaluation mechanisms usually emphasize the final outcome of learning activities, such as written reports or oral presentations. In our own EFL classroom context, it is not infrequent to find learners and instructors neglected the role of the L2 during classroom discussion or during some authentic collaborative dialogues. Previous studies reported that L2 learners resorted mainly to their shared L1 or fragmental L2 during these activities (Swain and Lapkin, 2000; Ortega, 2009). In our own classroom instruction, due to the learners’ learning culture (Jin and Cortazzi, 1997, 2006, 2017), we even observed that, sometimes, learners did not exchange ideas at all; instead, they simply wrote down the script in English and recited it for the oral presentation. However, reciting what is memorized as output is not natural production of language. Towell et al. (1996) observed that, compared with procedural knowledge, declarative knowledge requires more attention of the speaker, so less of it can be processed at one time than procedural knowledge. Therefore, in the writing-reciting process, where less spontaneity is required, the declarative knowledge stored in learners’ memory might be enough to support the output. If not, learners could still consult reference materials; whereas in authentic oral communication or collaborative dialogues, when immediate respondence is required, we assume that some EFL learners are unable to articulate their ideas may due to the slow processing of their declarative knowledge.

Given that it is during the planning and organizing process of learner-centered activities that learners could virtually interact and negotiate with each other in the L2 to facilitate the transformation of L2 declarative knowledge into procedural knowledge, to ensure EFL learners’ L2 use in collaborative dialogues, Swain and Lapkin (2000) as well as Cai and Zhang (2016) tried instructor monitoring to remind the learners to use the L2. However, we noticed a strong tendency among the learners to shift from their L2 to their L1 when the instructor moved away from their group. To tackle this problem, we decided to use the recording function of mobile phones to supervise the learners’ consistent use of the L2 without barring them from L1 thinking or bilingual digital dictionary consultation in the learning process. Various reports have shown that mobile-assisted language learning (MALL) facilitated L2 learners’ learning for various purposes (e.g., Mompean and Fouz-González, 2016; Xu et al., 2017; Lee et al., 2019). However, as Burston (2014) commented, studies on MALL’s application for monitoring learning remains rare. Although Carless (2007) reported that one teacher tried to motivate or check the learners’ use of the L2 by placing recording devices next to them, there have been no further studies on the effects of the approach. To fill this gap, the research reported in this paper, as part of a larger study, aims to investigate the effects of mobile-supervised QDCD on a group of lower-intermediate EFL participants’ academic oral English performance. It intends to find out whether the action of mobile phone-supported supervision would decrease the tendency of L1 use among the participants and whether it would enhance their oral English performance. This study also investigated whether the limited use of the L1-based SC-CSs would debilitate task accomplishment. Based on the research results, implications of MALL platform applications are discussed.

In studies on L2 oral development, researchers usually collect and analyze monolingual L2 data. Few studies worked on fragmental bilingual data, which are not regarded as an indicator of strong oral proficiency. However, when examining how L2 learners’ oral performance develops from a low level to a higher level, analyzing their broken L2 oral output becomes meaningful. Nevertheless, some of the existent measurements on complexity, accuracy, and fluency (Skehan, 1998; Housen and Kuiken, 2009; De Jong, 2016) are not quite applicable to fragmental bilingual oral data with high standard deviations, which usually feature the oral output of L2 learners with a low oral proficiency. As a result, we propose several adapted indices. Details are presented in the sections “Review of the literature” and “Oral performance measurement in this study.” As comparing L1/L2 word total was found to be problematic, this study adopts L1/L2 SC-CS analysis for language use tendency analysis, as explained in the Review of the literature section and the section on L1/L2 use tendency measurement in this study.

## Review of the literature

### L1 vs. L2

Traditionally, researchers, influenced by Krashen’s (1981) Acquisition-Learning Hypothesis, which suggests that acquiring a foreign language requires meaningful interaction in the target language, posited that teaching entirely through an L2 may help to develop learners’ in-built language system because it makes the language real, as pointed out by Macaro (2001). Some scholars warned that using the L1 in L2 classrooms inevitably cuts down on exposure to the L2 and might interfere in L2 development (e.g., Turnbull and Arnett, 2002; Howatt and Widdowson, 2004; Scott and de la Fuente, 2008). According to Anderson (1983), to convert declarative knowledge into procedural knowledge, learners go through three necessary stages: cognitive, associative, and autonomous. In the first stage, knowledge is stored as declarative and is retrieved slowly through interpretive mechanisms. In the second stage, speakers could get access to knowledge faster but the process is slowed down as they still need to refer to declarative knowledge from time to time. In the third stage, declarative knowledge becomes unnecessary and has developed into full procedural knowledge. Towell et al.’s (1996) research suggests that learners’ use of the target language is helpful to proceduralize their stored declarative L2 knowledge and achieve its automatization (see also Zhang and Zhang, 2020).

Since the turn of the century, however, an increasing number of studies have shown that teachers regard the use of an L1 in the L2 classroom as a helpful and constructive tool for linguistic, managerial and social purposes (Macaro, 2018; Ong and Zhang, 2018; De la Fuente and Goldenberg, 2020). Learners also appreciate the use of their L1 in L2 classes because they perceive its helpfulness in learning a new language (e.g., Neokleous, 2016). A few studies have reported that L1 use may help to enhance L2 speaking (Behan et al., 1997; Swain and Lapkin, 2000; De la Fuente and Goldenberg, 2020). For instance, Behan et al. (1997) examined the L1 (English) use of French (L2) immersion leaners in a late immersion program. They found that non-monitored groups used more L1 than monitored groups when preparing for their oral presentations. The researchers later judged the presentations of the non-monitored groups to be better than those of the monitored groups, concluding that “L1 use can both support and enhance L2 development, functioning simultaneously as an effective tool for dealing with cognitively demanding content” (p. 41). Undoubtedly, such studies defended the argument that L1 use could help to enhance immediate task completions, but their research results can not sufficiently support the conclusion that L1 use can enhance L2 development in the long run. De la Fuente and Goldenberg (2020) studied the L1-use effect on L2 learners’ writing and speaking proficiency development. They found that Spanish beginners who were allowed to use their L1 outperformed those who were not, both in speaking and writing. In their study, L1 was used extensively in teacher-learner, learner-teacher and learner-learner interactions for explicit grammar teaching and studying, vocabulary comprehension, task management, course policies explanation, classroom pedagogical approach discussion, learning strategies development, (inter) cultural knowledge study, and interpersonal relations. Yet the long-term effect of frequent L1-use during learner-learner interactions on L2 oral development among EFL learners of intermediate, upper-intermediate and advanced proficiency levels still remains unknown.

For people who have not personally experienced the challenge of learning English as a foreign language in a context where the language is not used in society, it may not be easy to understand why limiting the use of their L1 in classroom interactions is so critical. As is well known, it is not uncommon to find that many EFL learners, after studying the foreign language for 6–8 years or even longer, are still not able to use English to communicate orally although they could get quite high scores in reading, writing and listening on the TOEFL or the IELTS (Xu T. S. et al., 2022, 2023; Xu Z. Q. et al., 2022).

However, when foreign language learners shared a common L1 and when their L2 proficiency was low, the L1 tended to dominate in oral interactions in the foreign language classroom (Swain and Lapkin, 2000; Ortega, 2009; Savaşçı, 2014; Macaro, 2018; Ong and Zhang, 2018). Swain (1988) discovered that in classroom interactions, learners averaged two turns of talking per minute and 44% of the talk turns only produced one to two words each time. Merely 14% of the interactional time was found to produce extended discourse, utterances containing more than one clause, which is crucial for language development. We have noticed similar problems in our context among EFL learners, whose L2 oral proficiency is low, even though their written test results show that they have reached the intermediate level.

The phenomenon that “willing learners in an ESL setting who are unwilling to speak English within and beyond the boundaries of the classroom is not a trivial matter” (see Savaşçı, 2014, p. 2682). Such a problem exists among L2 learners from beginner to more advanced levels. Trying to tackle with learners’ L2-use reluctance problem, teachers have tried various approaches. In Carless’ (2007) study, L2 teachers reported how they had tried to encourage learners to use the L2: (1) walking around and reminding the learners; (2) encouraging learners not to be nervous in L2 speaking; (3) giving incentives, such as rewarding L2-use learners with stickers, stamps, or involving the learners in group competitions to show appreciation of L2-use; (4) appointing ‘language monitors’ by asking individual learners to try to remind their classmates to use English; (5) adopting whole-class tasks by asking the learners to come forward to the front of the classroom to use the L2; and interestingly, (6) motivating or checking learners’ use of L2 by placing recording devices next to the learner groups. However, the effectiveness of such methods was not discussed in detail in the research.

Although the L2-use reluctance problem among L2 learners has been long identified, few studies have aimed at the in-depth investigations into effective methods to prevent dominant L1 oral output in collaborative tasks. L2-use reluctance could cause pedagogical impediment if we over-interpret Swain and Lapkin’s (2000) caution that without the assistance of the L1 the learners may not have been able to accomplish the tasks as effectively (see also Macaro and Pun, 2018). We assume that, different from beginners, for EFL learners of lower-intermediate and higher proficiency levels, their L2 could be the dominant language used orally in the language classroom, while their L1 could assist them in other ways. Thus, this study, without prohibiting L1 use for thought assistance or digital dictionary consultation, was designed to examine how predominant L2 oral output in classroom collaborative dialogues would affect participants’ task accomplishment and their L2 oral performance in a 5-month academic intervention program. To encourage dominant L2 use, we perceived that some mobile devices for recording might be facilitating, and we intended to study if it would be the case.

### Studies on MALL

MALL refers to any language-related learning activities facilitated by mobile devices, which may include, but not limited to, smartphones, personal digital assistants, iPods, mobile digital recorders (or recording pens) and so on. Earlier implementation of MALL usually used mobile devices to deliver content, give vocabulary instruction *via* SMS (Yang, 2013) or MALL featured teacher-learner communication. Teachers seldom used the devices with an explicitly statement purpose or aim of encouraging learners to communicate (Kukulska-Hulme and Shield, 2008). Recent studies on MALL have extended to learner-learner and learner-teacher interactions in L2 learning (e.g., Al-Jarf, 2012; Moreno and Vermeulen, 2015; Schenker and Kraemer, 2017; Andujar and Salaberri-Ramiro, 2021). Sherine et al. (2020) studied the impact of WhatsApp interaction on improving L2 speaking skills by providing learners with various types of tasks through WhatsApp, such as filling in blanks, engaging in topic discussion through text-chat, interview, grammar test, among others. Phetsut and Waemusa (2022) studied how teachers’ feedback on L2 learners oral exercise through WhatsApp helped learners to improve their oral accuracy. Tseng et al. (2022) examined the overall average effect of mobile devices on L2 pronunciation learning by drawing on a meta-analytic framework. Their research results showed that the use of mobile devices had a significant effect on L2 pronunciation learning.

Notwithstanding the rich application of mobile devices in language learning, MALL tutorial applications, just as Burston (2014) commented, still face the pedagogical challenge of monitoring because instructors’ monitoring of learner performance, apart from providing learning outcome testing, remains rare. Hence the study on how mobile-supervised collaborative dialogues may influence the L2 oral performance of L2 learners embodies research significance.

### Studies on oral performance

Oral proficiency or performance is usually measured by complexity, accuracy, and fluency (Skehan, 1998; Housen and Kuiken, 2009; De Jong, 2016). A range of indices have been proposed or adopted for L2 oral proficiency analysis by different researchers. They include: (1) Temporal measurements: speech rate, average length of pause, mean length of run (MLR), etc. (e.g., Towell et al., 1996; De Jong, 2016); (2) performing measurements: different repair types (repetition, reformulation, replacement, false start and hesitation), number of repairs per 60 s (Tavakoli et al., 2020); (3) content measurements: ratio of reported necessary events to total necessary events (Trabasso et al., 1984); and (4) linguistic measurements: the ratio of error-free T-units and subordinate-clauses per T-unit, etc. (e.g., Foster and Skehan, 1996; Ong and Zhang, 2010; Rahimi and Zhang, 2018, 2019; Xu T. S. et al., 2022; Xu Z. Q. et al., 2022).

These criteria are generally more applicable to monolingual data analysis. Some of them are unsuitable for the analysis of L2 learners’ bilingual oral output. For instance, when the standard deviation of the total oral output is high, non-repeated words could describe oral performance more accurately than the measurements of repeated words, or repetitions per 60 s. For instance, if Learner A utters just five L2 words in 60 s without repetition, his repetitions per 60 s would be zero. In contrast, if Learner B uttered 40 L2 words with six repetitions in 60 s, his repetitions per 60 s reach six. Measured by repetitions per 60 s, Learner A outperformed Learner B, it seems. However, as we can see, Learner B, who produced 40 L2 words, was much more productive than Learner A, who only produced five L2 words during the same time span. In this case, Learner B demonstrated higher L2 oral proficiency than Learner A. For the same reason, calculating the total number of T-units could be more accurate than calculating T-unit per 100 syllables (Cai and Zhang, 2016).

### L1/L2 use tendency indicators

In many previous studies, while examining learners’ oral output quantity, total L2 words were calculated (e.g., Huang, 2010). However, for bilingual data, it is not able to tell whether the learners’ low L2 oral output results from their lack of ideas or from their L1 dominated expressions. In our previous study, we calculated the number of both the L1 and the L2 (see Cai and Wei, 2013) However, by doing so, we were unable to see what particular strategies the learners had used (e.g., whether they used the L2 for direct appeal or they reduced the form of the L2 by ignoring the grammar). In addition, a Mandarin word may contain one, two or four characters, for example, the English word “beautiful” could be “美,” “美丽” or “美丽动人” in Mandarin. If we calculate the Chinese characters, the danger of incomparability of the L1 and L2 data could also arise when the number of expressions in both language increases. Furthermore, calculating the Chinese words would be very complicated.

While communication strategies (CSs) are usually used to describe strategies used by L2 learners in oral communication, they could be classified into L1-based and L2-based CSs. Learners’ choice between L1-based strategies (e.g., code-switching, foreignization) and L2-based strategies (e.g., paraphrasing, restructuring, formal reduction) may reflect their confidence and tendency in L1/L2 language choice. Therefore, analyzing L1/L2-based CSs has a potential to demonstrate L2 learners’ L1/L2 use tendency and how they use them. However, up to date, there has been no such report. To fill this gap, we intend to study on how mobile supervision would affect L2 learners’ L1/L2 use tendency by analyzing their L1/L2-based CSs preference.

Dörnyei and Scott (1997) distinguished problem management CSs from problem-solving CSs (also see Hung and Higgins, 2016). Problem-management CSs are used during the language planning stage; while problem-solving CSs, such as meaning-negotiation and repair mechanisms (e.g., requesting and providing clarification), are used to handle problems that have already appeared in communication. Hung and Higgins (2016) identified six types of CSs, including interactional CSs, compensatory CSs, reduction CSs, focus-on-form CSs, sociocultural CSs and paralinguistic CS (i.e., mime). We can see that among the aforementioned CSs, some are used by speakers (e.g., providing clarification) some are used by listeners (e.g., requesting clarification); while some are associated with the language (e.g., focus-on-form strategies), and others are concerned with social or cultural interactions (e.g., sociocultural strategies; Zhang, 2010).

Among the various CS taxonomies, some inconsistencies or overlaps exist. For instance, in Tarone’s (1981) typology, “avoidance” either refers to “topic avoidance” (a learner resorts to abandon the concepts for which the L2 item or structure is not known) or refers to “message abandonment” (the learner, after starting to talk about a concept, stops in “mid-utterance” and is unable to continue). Such “avoidance” and “abandonment” are associated with the content of oral expressions. However, in the CS typology proposed by Faerch and Kasper (1983) “message abandonment” is categorized under “reduction strategies”，which also include “formal reduction” (phonological, morphological, syntactic and lexical). The terms “avoidance,” “reduction,” and “abandonment,” in different typologies, range from semantic category to topic areas, from phonological to lexical, from verbal to non-verbal ones.

To analyze L1/L2 use tendency of the intermediate-level EFL learners with low oral proficiency, proper CSs need to be selected, and analysis methods need to be figured out. This study intends to address these issues.

## The study

Our study aims to investigate how the recording function of mobile phones could be used to supervise L2 learners’ oral output, and how it could affect their L1/L2 use tendency and their academic oral L2 performance. Mobile phones were adopted in this research because it was a handy tool that almost every learner and instructor had at the university where data were collected. In other contexts, people could just as well use other mobile devices with recording functions. We chose the word “supervise” over “monitor,” because in L2 learning research, “monitor” implies the ability to detect errors and correct them (Krashen, 1981). However, the supervision function of mobile phones in our study was just to record the participants’ collaborative dialogues for the teachers to evaluate so as to encourage their L2 use.

Lowman (2000) warned that, without teachers’ sufficient guidance, classroom discussion often resulted in sacrifices not only in valuable classroom time but also in the attainment of lesson objectives. Cai and Zhang (2016) suggested that Question-Driven Group Discussions (QDGD) be used to enhance group interactive efficiency in EFL classroom instructions. In QDGD, EFL learners were guided by a sequence of text-based or content-based questions through their collaborative dialogues. They were required to negotiate the solutions to each question, write down the answers and hand them in within the given time. Besides being convenient for teachers’ evaluation, the written report was required also to ensure that the learners had reached a consensus on the solutions. According to Duff (1986), convergent tasks, solved together by learners, generate considerably more meaning negotiations than divergent tasks. Interaction research has shown that meaning negotiation could facilitate L2 learning processes (Mackey et al., 2007; Oliver, 2009; Mayo and Hidalgo, 2017).

As discussions often involve open-ended questions, we renamed QDGD as QDCD, because collaborative dialogues are those used by speakers in problem-solving and knowledge-building (Swain and Lapkin, 2000; see also Li et al., 2020), which may also involve closed-ended questions. In the current study, most questions were semi-open-ended or closed-ended. Although open-ended questions provide learners with the opportunity to communicate the reasoning process (Cooney et al., 2004), and thus they have the potential to elicit more extended discourses than closed-ended questions, our pilot study in an EFL context showed that, given an open-ended question, only a few learners would simply summarize their general ideas with a few sentences. Then the rest of the learners would express their appreciation of their partners’ ideas and the discussion ended without elaboration. Furthermore, open-ended questions give learners much freedom in thoughts as well as in choice of language, therefore, learners could easily evade unfamiliar ideas and expressions, which may debilitate L2 academic oral development (Cai and Zhang, 2016). As a result, we adopted semi-open-ended and closed-ended questions. In our current study we define a semi-open-ended question as a question which requires more than a short, fixed response, the respondent being expected to elaborate on the points to a certain extent. The acceptable variations of answers to the semi-open-ended question are confined by the context. In this study, the semi-open-ended questions are associated with the literature in the reading course, including paraphrasing, identifying implied meanings of sentences, appreciating writing techniques and so forth. Such questions have the potential of eliciting extended discourse, especially when learners are required to negotiate to render convergent solutions to the questions, but their opinions diverge owing to their differences in L2 proficiency, spectrum of knowledge, logical reasoning ability or personal experience, among other things.

To supervise L2 use during QDCDs, we used the recording functions of the mobile phones to record the participants’ collaborative dialogues. The mobile phone functioned as a supervisor, encouraging maximal L2 use without excluding the L1, as the participants could think in their L1, use bilingual digital dictionaries and even use some oral L1 when they really had trouble to express themselves in the L2. Mobile-supervised QDCDs was designed in accordance with the theory of Transdisciplinarity, which suggests that a successful integration of different disciplines should not result in more complexity and negate the comprehension of the involved parties but instead should make things easier to follow, and thus create a more effective learning environment (Colpaert, 2018).

## Research hypotheses

Drawing on the existing literature and related discussions above, we formulated two hypotheses regarding the effects of the mobile-supervised QDCDs on the EFL participants’ change in their L1/L2 use tendency by analyzing their L1-based and L2-based SC-CSs, and their academic oral performance in a naturalistic instructional environment:

*Hypothesis 1*: Mobile-supervised QDCDs will reduce the lower-intermediate-level EFL participants’ dependence on their L1-based SC-CSs in academic collaborative dialogues.

*Hypothesis 2*: Mobile-supervised QDCDs will help improve the lower-intermediate-level EFL participants’ academic L2 oral performance.

## Methods

**Figure 1:**
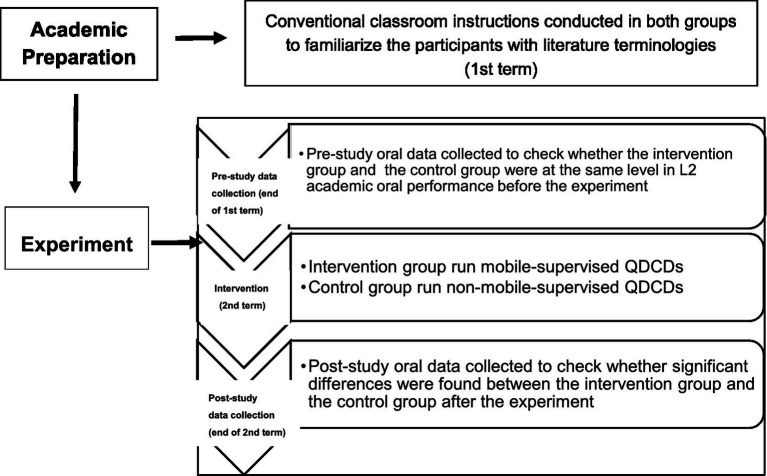
Research design and data collection flow.

The study was carried out in two classes of college juniors majoring in English. The general framework of the experiment is shown in Figure 1.

### Participants

The participants were a convenience sample of 50 university juniors majoring in English, ranging from 19 to 21 years old, from two different intact classes. They were two parallel classes in the same university, so they generally attended similar courses according to the syllabus. The two classes were randomly assigned to the intervention group or the control group. Before participating in this research, they had received at least 8 years of English education. All of them shared the same L1 (Mandarin Chinese). To make the data comparable, only the recordings of those who participated in both the pre-study recording and post-study recording were analyzed. Among 24 participants in the intervention group, three girls, who were the top students in class, were absent from the pre-study recording but attended the post one and one boy was absent from the post-study recording. Thus, there were 20 valid participants. Although these four participants were excluded from the analysis, the transcript of the three girls’ post-study recording is shown in Appendix 1. As the semester came to an end, the boy who missed the post-study recording, was not able to be recorded. All of the 26 participants from the control group participated in both the pre-and post-study recordings. 75% of the valid participants in the intervention group and 77% of those from the control group had passed the Test for English Majors – Band 4 (TEM-4), an examination set up by the State Education Commission of China in 1991 for intermediate-level EFL learners. It evaluates learners’ reading, writing and listening proficiency. Therefore, before the intervention, the participants were intermediate level EFL learners in terms of reading, listening, and writing; but according to the pre-study recording held in this study, although both groups were able to write down their answers to the questions which intended to elicit the pre-study oral data in English, they had trouble having collaborative dialogues about them in their L2. This seemed to suggest that their L2 knowledge was not activated for oral communication. Consequently, we defined them as lower-intermediate-level L2 learners, whose L2 language knowledge had been stored as declarative knowledge but it had not been activated into procedural knowledge for speaking.

### Instruments

Up to our knowledge, there has not been a perfect method which may measure oral output accurately. Many studies on oral proficiency, although used the same content in their pre-post tests, and validity was justified, adopted the examiner scoring system. Although the examiners were trained, the subjective features of the evaluation were still unavoidable. Zhang and Wu (2001) pointed out that oral data elicit methods, such as learners’ describing a picture orally, are not authentic communication. They suggested that authentic communicative tasks, such as discussion or problem-solving tasks, are needed to evaluate oral performance of learners. Douglas and Selinker (1992) observed that field-specific oral proficiency tests may provide more useful information than general-purpose tests when we aim to make field-specific judgments of learners’ oral language performance. Enlightened by their study, we designed field-specific question lists to elicit pre-post-study oral data. The questions resembled those provided to the participants in class throughout the semester but the question types and numbers were kept the same between the two oral data elicit question lists with two closed-ended questions on figures of speech, one closed-ended question on (relative) pronouns and three semi-open-ended paraphrasing questions (see Appendix 2). The participants followed the same instruction: to discuss and then write down their answers on a piece of paper before they handed it to the teacher for evaluation. They were instructed explicitly to discuss the first three questions either in English or in Chinese, but the last three in English only. This was to evaluate the participants’ initiative in L2 use and their ability to use it under pressure; namely, we would like to see how the control group and the intervention group would be different after the intervention in collaborative dialogues when they had freedom to choose from the L1 and the L2 (by analyzing the recording of the first three questions), and when they were forced to use the L2 (by analyzing the recording of the last three questions).

A popular oral test practice is to use the same materials to elicit the pretest and posttest oral data (e.g., Yang et al., 2020), but Mayo and Hidalgo (2017) discovered that, with a 1-year interval between the two tests (using the same materials), the post-test elicited more L1 than the pretest. Therefore, we decided not to use the same text. However, to improve the reliability of the two field-specific oral elicit question lists in parallel forms, the following measures were taken to eliminate interfering factors such as genre differences, participants’ lack of background information, and complex and long texts. Firstly, authentic, famous English political speeches were chosen, the first one written by Winston Churchill and the second one by John F. Kennedy. Secondly, the parts of the texts to be discussed for the pre-post-study recordings were deliberately left out, while the remaining parts of the texts and background information were studied together during a two-contact-hour teacher-centered classroom discussion, respectively, before the pre-study recording and the post-study recording. The Statistical Package for the Social Sciences (SPSS 13.0) was used for data processing.

### Data collection procedures

Before the study, the pre-study oral data were collected from the intervention group and the control group. The data from the two groups were compared to see whether the participants’ L2 oral performance was at the same level before the intervention. After the performance similarity of the two groups was confirmed, the intervention was administered to the intervention group. After the intervention, the post-study oral data were collected and results were compared to see whether there were any statistically significant differences between the two groups. The detailed processes are as follows:

The intervention was carried out in the Advanced English Course, a comprehensive English course offered in many Chinese universities to junior English majors to enhance their reading, speaking, listening, writing and translating skills. However, the course was traditionally taught with a focus on grammar and vocabulary. In recent years, it has been undergoing reform to restore its original full-skill-practice purpose. Both groups were taught by the same instructor (the first author), using the same textbook (*Advanced English Coursebook*). The course ran for two academic semesters, each of which lasted 18 weeks, with an average of three classroom hours per week.

To prepare the participants academically for the study, during the first semester, both groups received the same bilingual conventional instructions and were frequently exposed to academic literary terms (theme, tone, genre, characterization, symbolism, etc.), rhetorical devices (metaphor, simile, synecdoche, metonymy, transferred epithet, etc.) and terms about syntactic functions (subject, predicate, object, attributive, adverbial clause, etc.). The L1 was used by both the instructor and participants in classes for vocabulary study, cultural background introduction and academic term illustration. In their last class of the first semester, the pre-study oral data were collected in their classroom, using the pre-study oral elicit questions to elicit the participants’ QDCDs, which were recorded. Before collecting the pre-study oral data, recording was exercised several times in both groups during their QDCD to familiarize them with the process of recording.

Throughout the second term, the two groups received the same general teaching processes， except that 1 h each week was devoted to mobile-supervised QDCDs in the intervention group, while the control group went through non-supervised QDCD, discussing the same questions. To obtain authentic recordings of the collaborative dialogues, it is important to ask the participants to keep their mobile phones in recording mode without stopping them until the end of their discussion. After class, the recordings from each group were sent to the instructor through QQ, a popular mobile phone social communication app widely used in China, offering various services including the exchange of audio, video and other types of files between individuals or in groups. Some recordings were sent to the instructor through e-mails or saved directly to the instructor’s lap top through flash drives when the participants preferred. The instructor in this study sometimes brought several mobile phones to record the students’ QDCD, which proved to be quite convenient, because no data transferring is necessary. The steps in using QDCDs in the Advanced English course are illustrated in Table 1.

**Table 1:** Classroom steps in using QDCD (supervised/non-supervised).

Steps for using QDCDs		Teacher action in organizing QDCDs
Step1		Before class, preparing a sequence of questions associated with the text to be learnt
Step 2		Before learners start QDCDs, organizing class discussion on background information and key words
Step 3	Non-supervised	(1) Dividing learners into groups of four to six to discuss the questions; (2) moving around to offer necessary assistance
	Mobile-supervised	(1) Dividing learners into groups of four to six to discuss the questions; (2) directing learners turn on recording function of the mobile phones; (3) moving around to offer necessary assistance
Step 4	Non-supervised	At the end of the class, collecting learners’ agreed solutions to the questions in writing for the teacher’s evaluation
	Mobile-supervised	At the end of the class (1) collecting learners’ agreed solutions to the questions in writing for the teacher’s evaluation; (2) collecting learners’ recordings for teacher’s evaluation

Given that wrong answers from a group member could trigger and elicit further meaning negotiation and extended discourse, only the quantity of the recorded L2 oral output was generally evaluated, regardless of the correctness of the answers. No detailed evaluation was made and no detailed feedback was given to the participants about the recordings. To help the control group avoid debilitating anxiety in the post-study recording, mobile phone recording was also used with them from time to time, but the participants were told that the recordings would not be evaluated but were merely used for research purposes, and they were asked to go on with their QDCD in the way they usually do during classroom discussions. It turned out that their usual way was, just as we had observed before, to talk about the questions mainly in their L1 and wrote the answers down in the L2. Consequently, the supervising function of the mobile phone was removed. In the last class of the second term, the post-study oral data were collected in their classroom with the post-study oral elicit questions to elicit the participants’ QDCDs, which were recorded. Group members of the pre-study recordings and post-study recordings were kept the same. To guarantee the quality of the recordings, the recording devices used in the pre-and post-study recordings were prepared by the instructor.

Before both of the pre-study and post-study oral data elicit recording, the participants from both groups were told that the recordings made in class would be used by the instructor in a research project only and not for evaluation, and their personal information will not be revealed in any form anywhere. By doing so, neither of the groups was put in the examination context, yet they knew that their recordings would be heard by the instructor later. They all expressed consent to this procedure. We did this because, during the experiment, the intervention group’s QDCDs in each class somewhat resembled oral tests because their recordings were evaluated and scored. However, this was not the case for the control group. Thus, if the participants were put in the pre-and the post-testing conditions, the control group might suffer from anxiety and that may affect the reliability of the data. Therefore, the data we collected were the authentic QDCD data in the naturalistic academic instructional environment. At the end of the intervention, as the participants were extremely busy preparing for final examinations, a brief meeting was held by the instructor to collect the general comments and suggestions from the participants orally. The meeting was held in Mandarin Chinese, their L1.

### L1/L2 use tendency measurement in this study

As we mentioned in the literature review, to measure L1/L2 use tendency in this research, counting the number of L1 and L2 words was nonapplicable. We decided to resort to the calculation of L1-based CSs and L2-based CSs. As reviewed above, some CSs are used by speakers and some are used by listeners; some are used to solve language problems by manipulating available language knowledge, and we call them compensatory CSs; while others are labeled sociocultural strategies due to their communicative purposes. Given the aims of this study, we intended to promote speaker compensatory communication strategies for measuring the participants’ L1/L2 use tendency.

We adopted the SC-CS typology, as reported in Cai and Zhang (2016). Specifications of the typology are shown below:

1. Achievement Communication Strategies (CSs)
  *Independent Achievement.*
    - L1-based strategies: code-switching, foreignization, literal translation.
    - L2-based strategies: paraphrasing, word coinage, restructuring, formal reduction (phonological, morphological, grammatical).
  *Dependent Achievement.*

Direct appeal (L1-based direct appeal; L2-based direct appeal)

1. Abandonment Communication Strategy
  *Message abandonment.*

This typology analyzed L1-based SC-CSs and L2-based SC-CSs. We proposed that any attempt to express an idea, either grammatically correct or incorrect, in an L1 or an L2, be deemed as an adoption of the achievement strategy (see Cai and Zhang, 2016). Therefore, in this typology, abandonment only refers to the drop of topics or ideas. Achievement and abandonment strategies are regarded as content indicators rather than formal indicators.

While calculating SC-CSs, code-switching encompasses switching both at intersentential and intrasentential levels. Each intrasentential code-switching was calculated. If Student A said: 我觉得 “by hard” 应该是来之不易的吧 — difficultly gained, difficultly obtained. *{I guess that “by hard” may mean that something is not easily gained* — *difficultly gained, difficultly obtained}*, we coded it as using code-switching twice. While for the consistent L1 expressions, each L1 T-unit was calculated as one instance of code-switching, because we think that the L2 learner, after each L1 T-unit utterance, gets a very good chance to switch back to the target language, but he/she gives up the target language and switches back to the L1 again. This could be a good indicator of the learner’s tendency in the choice of the languages in communication. For example, if Student B said: 他知道有这么一条消息。[code-switching1] 知道具体的消息是什么。[code-switching 2] 他就是，他的助手向他报告了德国入侵俄罗斯的这一则新闻。[code-switching3] *{He knew there was such a piece of news. He did not know the exact information. That is his assistant reported to him the news about the invasion of Germany into USSR.},* we coded it as using code-switching three times. We can see that, regarding code-switching calculations, Student B (with three instances of code-switching) has a stronger reliance on the L1 than Student A (with two instances of code-switching). This is more reasonable than calculating all three L1 T-units as one instance of code-switching.

We also distinguished direct appeal in SC-CSs from common questions. For example, “What does the sentence mean?” was considered as a common question; while “How to say ‘优越性*{priority}*’ in English?” was a direct appeal.

### Oral performance measurement in this study

As we mentioned in the literature review that this study was different from previous studies in that it focused on bilingual data with high standard deviation and low proficiency, and we found that many existent complexity, accuracy and fluency measurements not applicable. As a result, we adopted one index to measure the total number of non-repeated L2 words, one to count the L2 T-units and another one to measure fluency. Space constraints make it impossible for us to describe these in more detail. Complexity, accuracy and fluency development are discussed in another paper.

Considering the features of bilingual data (L1 and L2 mixed), and drawing from the various existing oral proficiency measurements, especially those proposed by Cai and Zhang (2016), we used the following L2 oral performance indices in the current study:

*Non-repeated words (NRW)*. It is calculated by subtracting the repeated L2 words from the total number of L2 words uttered. [Repetitions used as a rhetorical strategy are not regarded as repetition, for example “It is done again and again.”]
*T-unit count (TC)*. It refers to the total number of independent clauses or independent clauses with their dependent clauses (Sotillo, 2000).
*Mean length of run after pruning (MLRP).* It is calculated as an average number of non-repeated L2 syllables produced between two pauses.

Run-on sentences were counted as two T-units and sentence fragments were not calculated (Sotillo, 2000). By doing so, we intended to calculate the sum of the non-fragmental utterances by the participants, disregarding their correctness. All the T-units were counted in this research, including those that had been interrupted but finally completed and those that were not topic-related (e.g., “Please put your book away.”). We understand that in an authentic communication environment, interlocutors need to fulfil multiple communication purposes, such as catching their partners’ attention, passing on things to each other, among other things, which represent the natural flow of communication. Whether or not a learner is able to use the L2 consistently for various purposes could also indicate her/his L2 performance. Therefore, it is unnecessary to exclude such utterances from the analysis of collaborative dialogues.

In this study, fluency was measured by the mean length of run after pruning (MLRP). Each disfluent pause of 0.25 s and longer, each terminating interruption (an interruption causing the termination of the current utterance), each time of code switching, and each time of L1-based direct appeal were calculated as a pause. If a disfluent pause, a terminating interruption, a code switching or an L1-based direct appeal overlapped with each other, they were integrated into one pause when we calculated the MLRP. Long pauses (silence caused by meditation), which lasted for 20 s or longer were pruned. The reading of the texts or the reading of the questions, as well as the talking with the instructor for help were also pruned.

### Data analysis processes

The bilingual recordings were manually transcribed by an instructor of English with the assistance of two English-major learners. The transcribing and coding processes are explained in some detail in Appendix 3. Information about group members and the length of all recordings pruned is provided in Appendix 4. The transcription was checked for correctness and accuracy and later on verified by the first author of this paper due to the fact that the second author was based in Auckland, New Zealand. Participants from the intervention group were coded with letters, such as StA and StB; while those from the control group were coded with Arabic numbers, such as St1 and St2. If a certain participant’s voice was indistinguishable from another, the parties concerned were asked to help to distinguish them.

As we mentioned before, when we designed the pre-post oral data elicit questions, we intended to see how the control group and the intervention group would be different after the intervention in collaborative dialogues when they had the freedom to choose from the L1 and the L2 for oral output, and when they were forced to use the L2. However, preliminary data analysis showed that that the participants’ choices between L1 and L2 seemed to be more ascribed to their level of confidence in the L2 than to the directions given by the instructor. The control group dominantly resorted to the L1 throughout both recordings, while the intervention group mainly used the L1 in the pre-study recording, but predominantly resorted to the L2 throughout the post-study recording (see Appendices 5, 6). It seemed that after the intervention, the intervention group had gained confidence in the L2 so even when they were given the chance to use their L1, they still stuck to the L2; However, in contrast, the control group stuck to their L1, even when they were required to use the L2 only. Since little difference was identified in the participants’ use of L1/L2 while answering the first three or the last three questions, we decided to use the response data for all six questions together. Nevertheless, if we analyzed the complete recordings of each group, it would be difficult for us to maintain the validity of the fluency variables associated with the sum (T-unit count and non-repeated words count) when the pruned lengths of each recording ranged from 12 to 21 min. Some groups uttered fewer words both in the L1 and L2 than the others, probably due to their limited ideas about some of the questions. Besides, owing to the different levels of anxiety felt by the participants at the different stages of the collaborative dialogue, some groups used more L2 expressions at the beginning of the discussion while the others used more in the middle or near the end. Therefore, to extract, for example, the first 2-min recording from each group could also be problematic. Finally, we decided to extract the parts with the most L2 utterances (see Appendices 5, 6). By doing so, we could examine each group when it demonstrates its L2 oral performance in full capacity, because collaborative dialogues with the most L2 expressions might mean that the participants were at their best in understanding those questions and had most ideas to express and were most willing to express. If any group hardly resorted to any L1 expressions, such as the intervention groups in the post-study recording, the most fragmental parts were pruned.

After the pruning, the pre-study recording of Control Group 4 turned out to be the shortest of all the recordings, averaging ~2.5 min for each participant. Taking 2.5 min as an average length of output per person, we extracted the recordings from each group. For instance, there were four participants in Control Group1, so we extracted a 10-min recording with the most L2 expressions. The recordings extracted turned out to be predominantly related to the discussions over the semi-open-ended questions.

The bilingual transcriptions of the extracted recordings were coded by two experienced instructors independently for communication strategies and performance indices. Any disagreements were worked out through discussions until the two coders came to 100% agreement. The transcripts were firstly coded for SC-CSs. Then repetitions were replaced by the mark of #. Each pause of 0.25 s and above caused by disfluency and each end of the turn of talking were coded with @. Interruptions from the others which caused the termination of the utterances were also marked with @. All these codes were then checked for consistency by the two of us authors as a way of verifying their accuracy of the two coders’ coding. Then the utterances made by each participant were singled out for further calculation.

The following example demonstrates the coded post-study recording excerpt of St22 from the control group. These utterances originally scattered among the utterances made by all the group members. We singled them out and put them together for analysis^1^ (below each L1 expression the corresponding L2 translation is provided):

Excerpt 1

St22:Forbear 是什么意思呀? [csw-L1].

*{What does “forbear” mean?}*

St22:世界范围里，大家还没有解决这个问题。[csw-L1].

*{The problem has not been solved worldwide.}*

St22:看不懂。 [csw-L1].

*{I cannot understand.}.*

St22:嗯 [csw-L1]，bitter peace,

*{yeah.}*

St22:是不是来之不易的和平呀?[csw-L1] 就是经历 [csw-L1] hard 和

*{Does it mean the hard-won peace?} {That is after experiencing hard and*

bitter 以后才得到的和平。[csw-L1] 所以我们要要更加，就是因为为.

*bitter, people finally got the peace.} {Thus we should be more, should be more.*

了这种和平要更加注意。[csw-L1].

*careful (to maintain) this kind of peace.}*

St22:就是，我觉得是不是经历过战争以后我们才，[csw-L1]就是慢慢的这.

*{That is, I feel that it may mean that only after we experienced the war, we gradually.*

种性格就不去再去挑起战争了[csw-L1]。就锻炼了。[csw-L1].

*form this characteristics of not starting wars.} {That means “annealed.”}*

St22:disciplined 怎么讲? [csw-L1].

*{How should we explain “disciplined”?}*

St22:他知道这个和平夹杂着 [csw-L1] …… 是痛苦的欢乐吗? [csw-L1].

*{He knew that this peace were mingled with … pain and happiness?}*

St22:嗯，那个叫什么来着?[csw-L1] 居安思危吧? [csw-L1].

*{Yeah, what do we call that? Be prepared for danger in times of peace.}*

St22:就是经历过磨难以后我们才知道在和平时代自己要自律的那种吧?

*{That is to say through tribulation we learned that we need to develop self-discipline.*

[csw-L1] 因为这个和平我们知道是来之不易的。[csw-L1].

*{in peace, because we know the peace was hard-won.}*

St22:来之不易的怎么说? [csw-L1].

*{How can we say “来之不易” (hard-won)” in English?}*

St22:Human rights? 还是ancient heritage? @ [csw-L1].

*{or}.*

St22:Hard 不用翻了。[csw-L1] 就是hard。[csw-L1] 就直接写hard，把.

*{We do not have to translate < It should be paraphrase > “hard.” It is hard. Let us.*

bitter 翻译一下就行。[csw-L1].

*{just write down the word “hard,” and translate < It should be paraphrase > “bitter”}.*

St22:我觉得是ancient heritage. [csw-L1].

*{I think it is ancient heritage.}.*

St22:后面还有一个[csw-L1] which. 21页。[csw-L1].

*{There is still another one after the word “which.” On Page 21.}*

St22:就先看前面的 [csw-L1].

*{Let us handle the former one first.}*

St22:Oh, my God! @.

St22:The human rights that the nation had committed to us @ can.

not be achieved @. [frd-L2].

St22:好下一个。[csw-L1] Next question.是排比吗?[csw-L1].

*{Ok, the next one.} {Is it parallelism?}*

Parallel. @ [frd-L2].

St22:Repetition. 哦不对。[csw-L1].

*{Oh, it’s incorrect.}*

St22:Parallelism and @ alliteration @. [frd-L2].

Using three asterisks (***) to represent the L1, we finally retrieved the following L2 utterances of St22’s: Forbear *** bitter peace, *** hard *** bitter *** disciplined *** Human rights? *** ancient heritage? @ Hard *** # bitter *** ancient heritage. *** which. *** Oh, my God! @ The human rights that the nation had committed to us @ cannot be achieved @. *** Next question. ***?Parallel. @ Repetition. *** Parallelism and @ alliteration @.

In the above data, words like “Forbear” and “bitter peace” were not regarded as reading from the book, because the speaker integrated them into the idea being conveyed. According to our pause calculation criteria, St22 paused 20 times altogether while she uttered 68 L2 syllables. Therefore, her MLRP was 3.4. It means that, on average, St22 only produced 3.4 syllables each time when she tried to use the L2. She uttered 39 non-repeated L2 words in total, with one T-unit. Every participant’s singled out L2 expressions were calculated with MLRP, TC, NRW, and SC-CSs, and then the *t*-test was used for data processing for the two groups.

## Results

### Hypothesis 1: Mobile-supervised QDCDs reduce dependence on L1-based SC-CSs

As the dependent variables were normally distributed, *t*-tests were run. As shown in Table 2, the independent-samples *t*-tests on the pre-study recording data demonstrated that, before the intervention, no statistically significant difference was found between the two groups in SC-CSs (for the L1-based achievement SC-CSs, *t* = −0.867, *p* = 0.39; and for the L2-based achievement SC-CSs, *t* = 1.521, *p* = 0.136). Both groups depended largely on L1-based achievement SC-CSs, the mean frequency of which was 32.4 for the intervention group and was 38.58 for the control group. Abandonment and direct appeal strategies were seldom used.

**Table 2:** Results of *t-*tests, means and SDs for SC-CS variables (pre-post recordings; intervention group = 20 vs. control group = 26).

SC-CSs		Groups	Pre-Mean	Post-Mean	Pre-Rd	Post-Rd	Pre-Rd	Post-Rd	Pre-Rd sig	Post-Rd sig
					SD	SD	*t*	*t*	*p* (2-tailed)	
Abandonment		Intv. Group	0.65	0.85	0.813	1.04	−0.153	1.47	0.879	0.149
		Contr. Group	0.69	0.35	1.011	1.231				
Independent achievement	L1-based	Intv.Group	32.4	1.25	21.7	1.446	−0.867	−10.39	0.39	0.000
		Contr. Group	38.58	32.88	25.519	15.431				
	L2-based	Intv.Group	6.55	13.4	4.322	7.155	1.521	6.09	0.136	0.000
		Contr. Group	4.62	3.12	4.243	2.747				
Direct appeal		Intv. Group	0	0.15	0.000	0.366	−1.806	1.83	0.083	0.083
		Contr. Group	0.12	0	0.326	0.000				

However, after the intervention, statistically significant differences between the two groups in the use of the L1-based and the L2-based achievement SC-CSs were identified: For the L1-based SC-CSs, *t* = −10.39 and *p* = 0.000; for the L2-based SC-CSs, *t* = 6.09 and *p* = 0.000. The intervention group used significantly fewer L1-based SC-CSs (mean = 1.25) than the control group (mean = 32.88); while it used considerably more L2-based SC-CSs (mean = 13.40) than the control group (mean = 3.12). The abandonment and the direct appeal SC-CSs remained low. Such a result indicates that during the post-study recording, whenever SC-CSs were needed, the intervention group predominantly resorted to L2-based SC-CSs. Interestingly, among all the L2-based strategies, formal reduction was used predominantly in both groups before and after the intervention.

### Hypothesis 2: Mobile-supervised QDCDs helps improve academic L2 oral performance

Hypothesis 2 was explored by drawing upon the data presented in Table 3. The pre-study means of the two groups showed no statistically significant differences:for MLRP, *t* = 1.031, *p* = 0.308; for NRW, *t* = 1.343, *p* = 0.186; and for TC, *t* = −0.176, *p* = 0.861. This suggests that at the beginning of the intervention, the two groups were almost at the same level in terms of their L2 performance. The means of TC for either of the groups were no more than two T-units. Nevertheless, in the post-study recordings, statistically significant differences were observed in the three oral performance indices between the two groups: for MLRP, *t* = 8.245, *p* = 0.000; for NRW, *t* = 3.918, *p* = 0.001 and for TC, *t* = 6.418, *p* = 0.000. The Standard Deviation of NRW in the post-study recordings was exceptionally high (103.11) for the intervention group, though.

**Table 3:** Results of *t-*tests, means and SDs for oral performance variables (pre-post recordings; intervention group = 20 vs. control group = 26).

Measurements	Groups	Pre-Rd	Post-Rd	Pre-Rd	Post-Rd	Pre-Rd	Post-Rd	Pre-Rd sig	Post-Rd sig
		Mean	Mean	SD	SD	*t*	*t*	*p* (2-tailed)	
MLRP	Intv. Group	3.17	5.23	0.595	1.078	1.031	8.245	0.308	0.000
	Ctr. Group	2.9	3.04	1.023	0.718				
NRW	Intv. Group	51.4	149.1	36.546	103.11	1.343	3.918	0.186	0.001
	Ctr. Group	36.96	49.15	35.854	55.64				
TC	Intv. Group	1.9	13.9	1.586	7.88	−0.176	6.418	0.861	0.000
	Ctr. Group	2	1.92	2.117	3.136				

## Discussion

### Changes in EFL participants’ SC-CSs use tendency

With respect to Hypothesis 1, which stated that mobile-supervised QDCDs will reduce the lower-intermediate-level EFL participants’ dependence on their L1-based SC-CSs in academic collaborative dialogues, the results clearly support this, as the excerpt from two participants randomly selected for illustration purposes here, StA from the intervention group and St26 from the control group, clearly illustrate.

Excerpt 1

**StA (pre-Rd):** 就是没区别。indistinguishable 就是没区别是吧?是一样的。.

*{It means there was no difference. Indistinguishable means no difference, does not it?}*

它那个，对，它是双重否定，是吧?Lo- @ lo- @ lo-那个词，loti- @ lotis-.

*{It is, yeah, it is双重否定 < litotes or double negation>, is not it Lo–lo- lo-?} {That word, loti-lotis-}.*

黑板上写着呢。 然后第三个, 老赵，.

*{It is written on the blackboard. Then the third one. Old Zhao < addressing her partner>.*

F. O. @ F. O. 是Foreign Office …

*{F. O. @ F. O. is Foreign Office}.*

***StA (post-Rd):*** The teacher has mentioned @ the same revolutionary belief is equal or equality. All men, all men are created equal @ that is endowed by their nature. “At issue” means revolutionary is still not solved. The revolutionary is objective. No. For them the focus is on the “belief” and “globe,” @ so it’s on the whole world not the independence of America. @The “revolutionary belief” means @ people @ all men are created equal…

***St26 (pre-Rd):*** 我觉得前面的I was awaken 还是没有I get up。.

*{I guess that the previously mentioned “I was awaken” means I did not get up yet.}.*

我觉得是。 to the effect which @ which said that @ Germany is @ likely to @ attack.

*{I feel it was so.}*

应该是has began to了吧?应该has take actions to @ to他听到了那个，他听到了.

*{It means “has began to,” does not it? Should “has take actions to to…* “*He heard that.*

攻击。意思是别人告诉他了吧?…

*{He heard about the attack. Does it mean someone told him?}*

**St26 (post-Rd)**: 祖先，就前面那些。yet 就是but 吧?But the @ revolution.

*{Forbears refer to the previously mentioned. Does “yet” mean “but”?}*

这要不要翻?不用翻? But the same revolutionary belief…@ 人生而平等。.

*{Do we need to translate it? No?} {All men are created equal.}*

啊!The revolutionary belief, @ such as the @ equality between @ humans @ human.

*{Oh!}*

being @ and 还有什么?就是一句话吗?到 global. 嗯，那就这句话，就这 …

*{What else? Just one sentence? Till “global.” Yeah, then it’s just this sentence, just this}.*

From Excerpt 2, we see that both participants resorted mainly to the L1-based code-switching strategy in the pre-study recording, but in the post-study recording StA used none of this, while St26 still stuck to it. We also observed that in the pre-study recordings, both participants resorted to the L1-based CSs even when they expressed very simple ideas, such as “是吧?” *{Is it?}* and “我觉得是” *{I think so.}*. During the early stage of the intervention, participants from both groups complained that speaking in the L2 required more attention and that interfered with their trains of thought; therefore, they preferred to use L1-based SC-CS. This might indicate that, before the intervention, for both groups, the L2 had not been activated as the language of communication.

In the post-study recordings, some participants from the control group still said: “我不能避免说中文 *{I cannot avoid speaking Chinese}*”; “快抓紧时间说中文，等会就不能说了 *{Let us make full use of the time speaking Chinese as we will not be allowed to do that later}*.” In contrast to this, the intervention group predominantly resorted to the L2 even when they were allowed to answer the first three questions bilingually. The intervention group reduced 60% their use of various SC-CSs, which was mainly replaced by successful direct L2 communication. Using the L2 became an initial and autonomous choice for them, suggesting that mobile-supervised QDCDs had unfettered the participants from their L1 dependence. Moreover, their task products (the answers to the questions in English in written format) were just as qualified as those of the control group. However, the control group, although they were provided with the same opportunities for collaborative dialogues, still heavily depended on L1-based SC-CS. As we mentioned before, the participants’ choices between the L1 and the L2 were more ascribed to their level of confidence in the L2 than to the directions given by the instructor. This discovery could explain, at least in part, why many L2 encouraging methods used by L2 instructors were unsuccessful as we mentioned in the literature review. This finding also implies that it is necessary and important to find methods which may effectively force and assist the lower-intermediate-level EFL learners to use the L2, and mobile-supervised QDCD looms to be a good solution.

Regarding the functions of CSs, quite a number of studies found that CSs would only temporally improve communication, and their overuse would hinder vocabulary and syntax development in learners (e.g., Aston, 1986; Skehan, 1996). As mobile-supervised QDCDs could help the EFL participants reduce their dependence on SC-CSs while resorting to direct problem-free L2 expressions, we anticipated that it would help their vocabulary and syntax development and, in turn, improve their performance in oral communication.

### Changes in EFL participants’ oral performance

Regarding the second hypothesis that mobile-supervised QDCDs will help improve the lower-intermediate-level EFL participants’ L2 academic oral performance, our analysis of data gives us evidence in support of this hypothesis in terms of MLRP, NRW and TC.

Götz (2013) claimed that MLR is a good indicator of speakers’ fluency, as it shows the natural amount of speech the speaker utters between interruptions. Grosjean and Deschamps (1975) observed that for native speakers, the MLR ranges from 7.42 to 14.85 syllables. In the intervention group, two participants’ MLRP surpassed seven syllables and three reached over six syllables in the post-study recording, approaching the lowest MLR level of native speakers’. The average MLRP of the intervention group increased to 5.23 in the post-study recording whereas that of the control group was 3.04. It is noteworthy that, compared with their performance in the pre-study recording, 11 participants out of 26 in the control group decreased in MLRP in the post-study recording, while none from the intervention group did. Tavakoli et al. (2020) found that MLR could successfully distinguish different levels of L2 oral performance. Towell et al. (1996) observed that the increase in MLR primarily resulted from the proceduralization of various types of knowledge such as lexical phrases and syntax. The MLR was regarded as an indicator of automatization in language performance (e.g., Towell et al., 1996; Skehan, 2009).

As we can see, although the intervention group made statistically significant progress compared with the control group in MLRP, it has not reached autonomous stage. However, the progress is considerable, because it suggests that, after the intervention, the majority of the participants from the intervention group could get access to their L2 knowledge faster although the process was still slowed down as they still need to refer to declarative knowledge from time to time. This seems to imply that the intervention helped them to progress from the “cognitive” stage into the “associative” stage of language learning.

As regards Non-Repeated Words, the intervention group’s performance increased from 51.4 to 149.1 on average, but that of the control group increased from 36.96 to 49.15. Regarding TC, at the beginning of the intervention, both groups’ T-unit production was low. Five participants from the intervention group and seven from the control group produced zero T-units. It can be speculated that, if the participants always resort to fragmental L2 expressions, little progress will be made in their L2 development. After the intervention, the average T-unit production of the intervention group increased to 13.90. This can be viewed as evidence of how mobile-supervised QDCDs helped the EFL participants in syntax development.

The standard deviation of the intervention group’s performance in NRW was high in the post-study recording, reaching 103.11. Virtually the standard deviations of the intervention group in MLRP, NRW, and TC were all higher than those of the control group. Although some participants apparently benefitted much more than the others from the mobile-supervised QDCDs, the big participant performance difference did not undermine the conclusion that the intervention was effective in the EFL context where the current research was based. This conclusion is drawn on the fact that all participants from the intervention group benefited from the intervention significantly in one aspect or another (see Table 4). Who may benefit more from this intervention and the reasons behind it could be an interesting research question that requires further investigation.

**Table 4:** Oral performance change of participants from the intervention group.

Students	MLRP		NRW		TC	
	Pre-Rd	Post-Rd	Pre-Rd	Post-Rd	Pre-Rd	Post-Rd
StA	3.0	7.4	72	260	1	25
StB	4.0	4.5	12	83	0	10
StC	3.0	6.7	70	153	4	13
StD	2.3	7.3	14	434	1	30
StE	3.5	5.2	22	49	1	6
StF	2.6	4	42	151	1	15
StG	3.3	5.7	119	275	5	27
StH	2.7	5.4	15	28	0	5
StI	3.3	5.1	12	56	0	9
StJ	2.6	4.1	8	53	0	6
StK	4.6	6.1	66	186	3	18
StL	2.3	3.9	27	30	0	5
StM	3.1	4.4	39	173	4	18
StN	3.7	4.8	61	291	3	23
StO	4.0	6.6	60	121	2	13
StP	3.4	5.1	99	152	2	14
StQ	2.7	4.6	24	59	2	3
StR	3.3	4.8	133	141	4	13
StS	3.1	4.1	59	129	3	7
StT	2.8	4.8	74	158	2	18

Although in previous studies scholars have argued for the facilitating functions of the L1 oral output in L2 development, the findings of our study seemed to suggest that, for lower-intermediate-level EFL learners, the quantity of L1 oral output used during learner-learner collaborative dialogues by the leaners might be inversely related to their L2 oral proficiency development in the long run. During the first few weeks of the current intervention, the intervention group lagged behind the control group both in the quantity and quality of their written answers to the questions because the L2 communication caused some difficulties in meaning negotiation. However, before long, they not only caught up with the control group in their written reports but also outperformed them in their L2 oral output. Their constant and sustained use of the L2 facilitated their L2 oral performance without impeding their reading comprehension or problem solution capacity. These results seemed to suggest that lower-intermediate-level EFL learners could successfully accomplish their academic tasks without depending on their L1.

According to the data we collected at the end of the intervention in a brief meeting we held with the participants, about 77% of the participants from the control group complained about the waste of time in discussing the questions. St10, for example, from the control group complained that everyone had different ideas about a question and so they had to persuade each other for a long time. As they always talked in their L1, they made no progress in English. Over 80% of the participants from the control group agreed with him. When asked why they did not use English as their L2 in the discussion, they said they doubted their ability to make each other understand in it. In contrast to this, however, over 90% of the participants from the intervention group felt that they had made progress in L2 oral performance because they said that they had benefited from the intervention. StQ, for example, from the intervention group said that his English was poor so he had worried about the possibility to discuss the questions in English. However, it turned out to be fine even though sometimes he, like others in the group, showed a little bit of anxiety when they were not able to express their ideas quickly. Although he felt he only made some progress in oral English, he felt much more confident to use it now.

Hence, we see that in the control group, where the L2 was not being used during the collaborative dialogues, the participants experienced some impatience and anxiety because they felt the discussion was time-consuming and their L2 oral performance barely improved. In contrast, the intervention group felt that they were making progress in improving their L2 oral performance despite them having to undergo some kind of stress, or more accurately, a kind of facilitating anxiety that became the driver for their perseverance in achieving success or reaching out for excellence. The progress they made gave them some enjoyment in learning English and in a way fostered their interest in a growth mindset and willingness to communicate in the L2 (see Jin and Zhang, 2021; Zhang et al., 2022).

By integrating teacher evaluation of the participants’ L2 oral output process into the evaluation mechanism, mobile-supervised QDCDs also motivated the participants to engage in the L2 collaborative dialogues because it became personally relevant (Bower, 2019): their task scores became partially related to their oral interactions. As the oral output emphasized quantity over quality, and the L1 was still allowed for dictionary consultation and communication assistance, the level of difficulty of the tasks was reduced. The less demanding tasks helped the participants to overcome their doubts about their ability to communicate in the L2, and gradually, with the progress they made, their anticipated value of the outcomes of the activities increased, and therefore the participants were further motivated (Bower, 2019).

In our earlier work (Cai and Zhang, 2016; Zhang and Zhang, 2020), the design of the study was such that the instructor supervised the use of the L2 closely; and in the current study the mobile phone functioned as the supervisor. It can be surmised that in our 2016 intervention as well as in the one reported in this paper, consistent L2 use in the classroom appears to be crucial to the participants’ successful and sustainable oral performance in QDCDs.

### Implications and suggestions

Our research results seemed to suggest that predominant L2 use during the planning and organizing process of learner-centered activities for lower-intermediate-level EFL learners was quite effective in promoting their L2 academic oral performance; although predominant L1 use helped to enhance the learners’ immediate task completions, it seemed to have negative effects on their oral development in the long run. Such a conclusion was drawn from the research results that the control group which dominantly used the L1 during the experiment made little progress in oral performance at the end of the experiment although they outperformed the intervention group in answering the questions given by the teacher in the first few weeks. For lower-intermediate-level EFL learners, our findings seemed to support Krashen’s (1981) Acquisition-Learning Hypothesis, which suggests that acquiring a foreign language requires meaningful interaction in the target language and that teaching entirely through an L2 may help to develop learners’ in-built language system because dong so makes the language real, as pointed out by Macaro (2001). Although Behan et al. (1997) found that L1 use during preparations for their oral presentations promoted oral performance of the learners, such conclusion was mainly drawn from the evaluation of the learners’ immediate task products rather than on the evaluation of their oral performance in a longer term. We are of the view that for L2 beginners, L1 use could be facilitating in vocabulary and grammar learning; however, for more advanced learners, dominant L1 use may hinder their L2 oral language development, because the chance for L2 proceduralization is limited without authentic use of the language in real situations.

This study also investigated whether the limited use of the L1-based SC-CSs would debilitate task accomplishment. Although some researchers cautioned that without the assistance of the L1 the learners may not have been able to accomplish the tasks as effectively (Swain and Lapkin, 2000; Macaro and Pun, 2018), which can be true in some cases; however, we argue that we should not over-interpret such cautions by overemphasizing the role of the L1 in L2 learning. Our study found that while predominantly using the L2 during the collaborative dialogues, the intervention group, only in the first few weeks, lagged behind the control group in the quantity and quality of their question-answer tasks during QDCD, because the L2 communication caused some difficulties in meaning negotiation. However, before long, they were not only able to catch up with their peers in the control group in their written reports but also outperformed them in their L2 oral output.

Drawing on the above discussions, we suppose that, for lower-intermediate-level, intermediate level and high level L2 leaners, it might be advisable to integrate teacher evaluation of the participants’ L2 oral output during the planning and organizing process of learner-centered activities into the evaluation mechanism, and mobile-supervised QDCD could be facilitating to such operations. Mobile-supervised QDCD are applicable to many different types of student-centered language learning contexts, for instance, task-based instruction (TBI), project-based in struction (PBI), and content-based instruction (CBI).

While designing mobile-supervised QDCDs, it might be advisable that instructors observe several issues: Firstly, it might be necessary to find proper schemes to reduce the level of difficulty of the requirements. For instance, in our study, the oral recording evaluation emphasized quantity over quality, and the L1 was still allowed for dictionary consultation and communication assistance. In this case, the less demanding tasks helped the participants to overcome their doubts about their ability to communicate in the L2, and gradually, with the progress they made, their anticipated value of the outcomes of the activities increased, and therefore the participants were further motivated (Bower, 2019). Secondly, our research findings suggest that for mobile-supervised QDCDs, semi-open-ended questions might elicit more collaborative dialogues than open-ended and close-ended questions, because, in this experiment, the transcritions of the recordings extracted for data analysis with most collaborative dialogues turned out to be predominantly related to the discussions over the semi-open-ended questions. This might result from the features of such questions, which require more than a short, fixed response, the respondent being expected to elaborate on the points to a certain extent. Such questions have the potential of eliciting extended discourse, especially when learners are required to negotiate to render convergent solutions to the questions, because learners’ opinions could diverge owing to their differences in L2 proficiency, spectrum of knowledge, logical reasoning ability, or personal experience, among other things. Thirdly, the teacher-provided recording devices seemed to be more effective in promoting authentic collaborative dialogues than learner-owned devices because learners seemed to feel that they had no right to turn off the teachers’ recording devices when they attempted to write down their scripts before speaking.

As for oral output evaluation, there has not been a perfect method for measuring oral output accurately. Zhang and Wu (2001) suggested that authentic communicative tasks be used. Enlightened by the field-specific oral proficiency tests suggested by Douglas and Selinker (1992), we designed the field-specific question lists to elicit pre-post-study oral data (see Appendix 2). Field-specific questions are questions associated with the specific academic realm which the L2 learners are learning. These academic realms could be engineering, education, medication, language, agriculture, etc. By measuring the linguistic features of the oral data elicited from these questions, such as MLR, T-unit, etc., we are able to evaluate the academic oral performance of the learners. We consider this more accurate than the examiner scoring system which is often adopted in oral output evaluation. Field-specific question list might be used potentially as an academic oral test format, but its validity requires further study.

In this study, field-specific question lists were also used to elicit oral output among learners in QDCD. Under the supervision of mobile phones, they seemed to be quite effective. Such questions are associated with the academic realms that the learners are learning, and these topics might also be associated with their future academic evaluation or future career; therefore, the questions become personally relevant to the learners (Bower, 2019).

Our study also showed that fragmental oral output is worth our instructional attention because it helps us understand how L2 learners develop from low level to higher level oral performance. In the current study we suggested the methods of singling out an individual’s utterance from dialogues or group discussions for oral analysis, coding and analyzing bilingual fragmental oral outputs (see Appendix 3). However, their validity requires more investigations and additional methods for such data analysis require further study.

For the application of mobile-supervised QDCD, mobile recording devices are important. Although MALL usually refers to any language-related learning activities facilitated mainly by smartphones, personal digital assistants, iPods, we would like to take a more generalized concept of MALL. We hold that any mobile devices, such as mobile digital recorders (or recording pens), could be included as far as they are mobile and could facilitate language learning, as Colpaert (2018) pointed out, in his theory of Transdisciplinarity, that a successful integration of different disciplines should not result in more complexity and negate the comprehension of the involved parties but instead should make things easier to follow, and thus create a more effective learning environment. Compared with multi-media classrooms equipped with recording devices, mobile recording devices are more flexible and applicable as they may fit into diversified environments: in or outside classrooms, in developed areas where smartphones and the Internet are available, as well as in less developed areas where those devices are inaccessible but relatively cheap recording pens are available.

Our research results may also have implications for MALL platform applications. While many current MALL platforms, such as *Unipus* and *pigai.org* in our context, only provide to L2 learners with oral practice exercises such as reading-aloud practice, monologue or dialogue about daily issues (e.g., about hobbies, giving advice, etc.), our research results seem to suggest that it may be advisable to integrate QDCD academic exercises into the MALL platforms for more advanced EFL learners, where learners could record their L2 collaborative dialogues over certain field-specific questions and then upload them for teacher evaluation and feedback. This could largely enrich the realm of topics and enhance authenticity of communication in oral practice for intermediate-and-above level EFL learners.

### Limitations

Despite all the attention we gave to potential issues, we still think that a statement on some limitations is in order. Firstly, given the intensity of the study we had a relatively small sample size and the limited range of performance groups. Accordingly, readers need to exercise caution when interpreting the results. Secondly, instead of using the same materials, we designed two lists of field-specific oral data elicit questions associated with two different texts to elicit the pre-study and post-study oral data. Although we controlled the genre difference, participants’ lack of background information, text complexity and the length of the texts, and when compared with the commonly used oral test examiner scoring system, our linguistic analysis approaches (e.g., counting MLRT, TC and CSs) of the participants’ oral output may not be less objective if not more objective; the potential of using such question lists as oral tests still requires further study. Thirdly, interlocutor effects might have influenced the results of the research. In addition, we counted code-switching as pauses because code-switching might have caused interruptions to L2 utterances. When calculating L2 MLRP, we cannot overlook the possible interruptions caused by code-switching. Previous studies on bilingual oral data seldom measured MLR, maybe due to the difficulty in defining pauses. However, MLR is an important indicator of oral proficiency development, and it seems necessary to find proper ways to measure it when dealing with bilingual data. Therefore, the method used in our study requires further exploration. A further limitation is that in our analysis we extracted an average length of 2.5-min oral output per person from each 4-to-6-person group recording, in which most L2 expressions were uttered. The validity of such performance also needs to be verified through more thorough studies in similar contexts. Finally, we did not create the pretest and posttest conditions for the purpose of alleviating the control group from anxiety. Therefore, the data collected in the current research demonstrate the change that occurred among the participants in their authentic classroom academic oral interactions before and after the experiment. Whether the intervention could influence intermediate EFL learners’ overall academic oral proficiency also requires further study.

## Conclusion

This study was set up to examine whether mobile-supervised QDCDs would help the participants improve their L2 oral performance. Two hypotheses were examined: 1) mobile-supervised QDCDs will reduce the lower-intermediate-level EFL participants’ dependence on their L1-based SC-CSs in academic collaborative dialogues; and 2) mobile-supervised QDCDs will promote the lower-intermediate-level EFL participants’ L2 academic oral performance.

Having nothing to say and not using their L2 are two obstacles to lower-intermediate-level EFL learners’ L2 oral proficiency development. By using list of questions to offer scaffolding for collaborative dialogues and by using mobile-device supervision to motivate the participants to speak in their L2, the mobile-supervised QDCDs seemed to be effective in improving the participants’ L2 oral performance. The mobile-supervised QDCDs successfully elicited more oral L2 output from the EFL participants. It provided a reasonably good platform for the EFL participants to negotiate meaning in their L2. As a result, such collaborative dialogues helped them, statistically speaking, reduce their dependence on SC-CSs significantly, especially on those L1-based SC-CSs, and they improved their L2 oral performance in areas such as MLRP, NRW, and TC. These appeared to have helped the L2 participants proceduralize their stored declarative L2 knowledge and move toward automatization in the very process of L2 production. The research result somewhat relieves us from the worries that without L1 oral output, lower-intermediate-level EFL learners may not successfully accomplish their academic tasks.

From a pedagogical point of view, our results suggest that it is advisable for teacher evaluation of leaners’ L2 oral output process to be integrated into the evaluation mechanism so as to enhance their motivation in developing their L2 communicative abilities. Hence, they may have implications for the teaching and learning of EFL in other similar contexts. They might be able to shed light on the ongoing debate on whether interactions should be taken as an adjunct to conventional approaches or as an independent self-sufficient pedagogical methodology (Long and Crookes, 1992; Zhang and Zhang, 2020). Furthermore, QDCD might need to be integrated into MALL platforms to promote academic oral proficiency of intermediate and above level L2 learners.

Our findings also point to additional implications: (1) predominant L2 use during the planning and organizing process of learner-centered activities for lower-intermediate-level EFL learners seems to be quite effective in promoting their L2 academic oral performance; (2) question-guided field-specific oral data eliciting questions could be used potentially as an academic oral test format; (3) semi-open-ended questions, compared with open-ended and close-ended questions, tend to elicit more collaborative dialogues in QDCD; (4) fragmental oral output is worth our instructional attention because it helps us understand how L2 learners develop from low level to higher level oral performance; (5) methods in singling out an individual’s utterance from dialogues or group discussions for oral analysis are useful (see Appendix 3); (6) the methods for coding and analyzing bilingual fragmental oral outputs are relevant (see Appendix 3); and (7) L1/L2-based SC-CSs could be regarded as indicators of a language use tendency when calculating the L1/L2 word total is inapplicable.

Clearly, many issues related to mobile-supervised QDCDs require further investigation (e.g., appropriate methodologies to collect and analyze bilingual or multilingual oral data). Given the design of our study and its findings, future studies may need to investigate whether QDCDs would help EFL learners improve oral proficiency in terms of accuracy and complexity in oral expressions and the appropriate ways for their measurement. Future research might also want to include learners of a range of proficiency levels in order to examine the utility of QDCDs as a pedagogical tool for helping learners improve their L2 proficiency. Researchers interested in this line of research might also want to replicate this study in other foreign language teaching and learning contexts.

## Data availability statement

The original contributions presented in the study are included in the article/Supplementary material, further inquiries can be directed to the corresponding author.

## Ethics statement

The studies involving human participants were reviewed and approved by the School of Foreign Languages Ethics Review Committee at Huazhong Agricultural University, China. Written informed consent to participate in this study was provided by the patient/participants.

## Author contributions

YC and LZ conceived and designed the study. YC collected and analyzed the data and drafted the manuscript. LZ finalized it for submission as the corresponding author. All authors contributed to the article and approved the submitted version.

## Funding

This work was financially supported by a grant from the Teaching Reform Research Program of Shenzhen Technology University: Studies on Communication Strategies Used by College Students in ESP Classes. It is also part of the follow-up study of “A Study of the Learning Plateau Phenomenon in Oral English Development (project no. 2012ZYTS001)” sponsored by a grant from the Fundamental Research Funds for the Central Universities.

## Conflict of interest

The authors declare that the research was conducted in the absence of any commercial or financial relationships that could be construed as a potential conflict of interest.

## Publisher’s note

All claims expressed in this article are solely those of the authors and do not necessarily represent those of their affiliated organizations, or those of the publisher, the editors and the reviewers. Any product that may be evaluated in this article, or claim that may be made by its manufacturer, is not guaranteed or endorsed by the publisher.

## Footnotes

^1^Legends: ([csw-L1] = code switching <L1-based>; [frd-L2] = formal reduction <L2-based>; @ = pause; # = repetition; da-L1 = direct appeal<L1-based>).
