# Supplementary material for: Effects of mobile-supervised question-driven collaborative dialogues on EFL learners’ communication strategy use and academic oral English performance
Source: Front Psychol. 2023 Jul 3;14:1142651. doi: 10.3389/fpsyg.2023.1142651 (PMC10352317; doi:10.3389/fpsyg.2023.1142651)
Supplement: Supplementary file 1 [file Data_Sheet_1.docx]

**Appendix 1:** Post-study recording of 3 students who were absent from the pre-study recording

Gril 1: Uhr, the question I think the answer is @ antithesis, because the use of, uhr, @ “vic- victory” or “end”, it just, com-pared each other [contrast with each other]

Girl 2: And the second one. “Revolutionary belief” means the e-equality.

Girl 1: Ah, yes, nice. [Reading from the book]… “Forbears” is an-cestor. “Still at issue”, I think, ah, issue means it’s controversy, controversial around the global actually. I believe it to be .

Girl3: Yeah. To @ paraphrase it.

Girl 1: “The same revolutionary …” [reading from the book] I think it’s means that the @ belief, @ the equal belief which uhm … @ [giggling] equal belief which …@ uhm our ancestors fought fought for is still eh, controversial @ in the world. @ Here is the “yet” and the “saying”. I think it’s not enough to explain it [giggle] clearly, so you add something. 来，这句话… 就是后面的这句话。

Girl 3: You already explain the lines.

Girl 2: That’s the idea, I think.

Girl 1: Ok, say something [teasing and giggling]

Girl 3: …You already say it. What do you mean?

Girl 2: So we are done with the second reference?

Girl 1: Ok. The third one is … [reading]

Girl 3: Where is it? [reading from the book] @ 慷慨，悲惨？

Girl 2: Oh, you … think the @ meaning of disgrace similar with the “displace- dissuppline” {mispronunciation of “discipline”} ?

Girl 1: “Tempered by war”, it’s in 1961, I think it was … @

Girl 2: means that … @ Americans

Instructor: I guess you three will be the best group.

Girl 1: Why?

Instructor: The top students!

Girls: [giggling]

Girl 2: And the second one, we should organize our sentence. …

Girl 1: Means that …

Girl 2: You… What do you think about this one? You two? …

Girl 3: I think this, um, “tempered by the war or dis-disciplined by uh hard bitters bitter peace” means that Americans went through war and a lot of hardship. After that their, their characters are shaped.

Girl 1: Yes, I agree with you.

Girl 2: I’m writing it. “After …”’

Girl 1: After going through 吗？…

Girl 3: 行吧。

Girl 2: 对。

[ Long thinking]

Girl 3: The @ Here is the, this nation has always been committed to which …

Girl 2: It should be.

Girl 3: to which …

Girl 2: blablabla...

Girl 1: I mean the the sentence of this “human rights to which”, here here “to” is the part of “committed to “ , so I think “which” should be … [interrupted by the instructor] I think it just means uh, human rights?

Girl 3: Human rights.

Girl 2: This human rights.

Girl 3: Do you think “which” just refer to the a word, or a short sentence or a phrase?

Girl 1: [reading] I think, it it means Americans has the intention to to do some, uh, to take some measures to, uh, protect their human rights, but there are some obstacles, um, which, um, which .. you know ...

Girl 3: So there are “slow undoing of this human right” means Americ-American people at that time do not protect human rights.

Girl 1: Yes. Yes, I think the part of reason is because that there are a lot of war, which they should, they uh, they need take a lot of time and spend a lot of money and human resources to this, so “which” is think it it means human rights.

Girl 3: [reading]

Girl 1: Oh, that sentence.

Girl 2: “unwilling to witness or permit … the slow undoing …” [reading]

Girl 3: We don’t want to see or arise the, the, the gradually warning of the human rights. Um, um because the protection of the human rights is the, is the, um is the mission our nation has always been kipt [mispronunciation of kept] on.

Girl 1: 我这里有个，后面这个 “is their mission blablabla ...” [reading]

Girl 3: I think, this, this uh … @ sentences means that American people has undergone the war and at that time, no stop of the people are … have @ had that ability to @ to prevent the slow erosion or damaging this human right. Although, although America has always … [long pause] 就是他们那时候在经历战争嘛，

Girl 1: En.

Girl 3: So they have less able to protect, prevent the pra-practice of erosion, erosion or damaging this human right. Although this need America has always devoted to this this flo-float boots [mispronunciation]

Girl 2: 是不是America 崇尚什么呀？

Girl 1: Uh, uh.

Girl 3： Loft, loft course.

Girl 2: Aha, 我来看一下。

Girl 3：loft 是的，loft course.

Girl 1: American people …

Girl 3: Lofty, lofty course.

[writing down the answer]

…

**Appendix 2** Pre-Post-Study oral elicit question lists

***Pre-study oral data elicit questions.*** It is composed of six questions, as shown below:

Please discuss the following questions with your group members and write down the consensus answers on a piece of paper in English and hand it in at the end of the class. **Questions 1-3** could be discussed **either in Chinese or in English**, but **Questions 4-6** are to be discussed **in English Only**.

1. Paraphrase this sentence: “I was awoken at 4 a. m. the following morning by a telephone message from the F. O. to the effect that Germany had attacked Russia.”
2. What figure of speech is used in the sentence “The Nazi regime is indistinguishable from the …”?
3. Paraphrase the sentence “It is devoid of all theme and principle except appetite and racial domination.”
4. What figures of speech are used in the sentence “No one has been a more consistent opponent of …”?
5. What does “it” indicate in the sentence “I will unsay no word that I have spoken about it”?
6. Paraphrase the sentence “The past, with its crimes, its follies, and its tragedies, flashes away.” (*based on the excerpt from “Hitler’s Invasion of U.S.S.R.” by Winston Churchill*)

***Post-study oral data elicit questions.*** It consisted of six questions, which are stated in detail below:

Please discuss the following questions with your group members and write down the consensus answers on a piece of paper in English and hand it in at the end of the class. **Questions 1-3** could be discussed **either in Chinese or in English**, but **Questions 4-6** are to be discussed **in English Only**.

1. What figure of speech is used in the sentence “… we observe today not a victory of party, but a celebration of freedom — symbolizing an end, as well as a beginning — signifying renewal, as well as change”?
2. Please paraphrase this sentence: “For man holds in his mortal hands the power to abolish all forms of human poverty and all forms of human life.”
3. Please paraphrase the underlined part of this sentence: “Let the word go forth from this time and place, to friend and foe alike, that the torch has been passed to a new generation of Americans — born in this century, tempered by war, disciplined by a hard and bitter peace, proud of our ancient heritage ...”
4. What does “which” indicate in this part of the sentence “unwilling to witness or permit the slow undoing of those human rights to which this Nation has always been committed”?
5. What figures of speech is used in “… that we shall pay any price, bear any burden, meet any hardship, support any friend, oppose any foe …”?
6. Please paraphrase the underlined part of this sentence: “Let every nation know, whether it wishes us well or ill, that we shall pay any price, bear any burden, meet any hardship ...” (*based on the excerpt from “The Inaugural Address” by John F. Kennedy*)

**Appendix 3:** Transcribing and coding processes

Step 1: Transcribe the recordings from each group verbatim (for examples, see Appendixes 5-6)

Step 2: Marking intrasentential and intersentential pauses with @

(could be done simultaneously with Step 1)

Step 3: Code SC-CSs

Step 4: mark each turn as a pause with @

Step 5: select the part with most L2 utterances

(Long pauses over 20 seconds pruned. for examples, see Appendixes 5-6)

Step 6: single out each group member’s utterances by deleting the utterances of the others

Step 7: replace each cold-switching (L1 utterance: phrases or sentences) with “***”

Step 8: mark the repetitions with #

Stept9: calculate non-repeated L2 words, each type of SC-CSs and pauses

Step10: calculate pauses (If a disfluent pause, a terminating interruption, a code switching or an L1-based direct appeal juxtaposed each other, calculate them as one pause)

Step11: calculate MLRP

**Appendix 4:** Length of all original recordings (pre- post recordings)

*[Note: The following length of recordings was obtained after the long pauses caused by meditation (20 seconds and above), the talking with the instructor for help and the reading from the book had been pruned.]*

| **Groups** | | **Pre-Rd** | **Post-Rd** |
| --- | --- | --- | --- |
| Control  Group | Group1 | 12’27“ (4 students) | 17’02” （4 students） |
|  | Group2 | 12’30” (5 students) | 21’51” (5 students) |
|  | Group3 | 12‘37“ (5 students) | 19’42” (5 students) |
|  | Group4 | 14’48“ (6 students) | 18’11” (6 students) |
|  | Group5 | 15’02” (6 students) | 18’30” (6 students) |
| Intervention  Group | Group1 | 13’22” (5 students) | 14’57” (5 students) |
|  | Group2 | 15‘22“ (6 students) | 17’13” (6 students) |
|  | Group3 | 12‘51” (5 students) | 20‘57” (5 students) |
|  | Group4 | 12’40” (5 students) | 13’48” (4 students + 1 absent) |
|  | Group5 | 0’ (3 students absent) | 12’ (3 students) |

**Appendix 5:** Post-study recording transcript of Intervention Group 4

*[Notes: 1) Take a Letter from the name of a student to represent the student to avoid confusion. Later use the alphabetic letter to replace the name;*

*2) select the part with most L2 utterances;*

*3) 4 learners in the group so select 10 minutes of the recording*

*4) intersentential pauses are marked during transcription; pauses at the end of each turn were marked later.*

*5) The following total recording time length of each group was obtained after the long pauses caused by meditation (20 seconds and above), the talking with the instructor for help and the reading from the book had been pruned. ]*

*Total voiced recording time* : 13’48’’

**time span 0’20”- 1’14” [deleted]**

F : always hold a @, is still a @

T: World issues. @

F : Controversy 争论 around the world. @

T: Around the world means the worldwide issues. @

F : Ye-yes. @

T: The cri- 那啥，the forebears fought指啥？@【Reading from the book】

F : Forebear 的解释是four three bears. @

T: Means what ? @

L: Forebears? @

F : Ancestor., Ancestors. @

T: fought? @

L: Straigle. @

T: Struggle. @

F : The struggle is still a worldwide issue. @

T: Problem 应该是。ok. @

**time span 1‘15”-2‘04 [selected]**

T: But, @ up to now. @ Up to now, the @ the forth… @

F : the faith. @

L: the faith. @

H: faith. @

L: Faith we hold. @

F : What kind of faith? @

T: The faith we …. @

F : What kind of faith? @

T: For equality. @

F : The faith for equality @ that our ancestors struggle for. @

【wring the answers】

**time span 2’35”-2’52 [selected]**

F : At issues means is still @ controversy. 争论的意思 @

L: A worldwide issue. @

F : Worldwide issue 也可以。@

T: worldwide problem. @

F : Worldwide controversory problems. @

【Reading, thinking】

**time span 4’33”- 4’50 [selected]**

F : Tempered is a new word. @

H: Temper-ed @

F :　Temptation means? @

T: Attraction. @

F : Lured by. @

**time span [deleted: 4’51-5’15” delete 24“]**

T: Temper means 脾气。@

H: Temper 动词吧这不是？@

Tempted, tempted 是名词。@

H: 动词就是缓和啊。@

L: soft. @

【reading】

**time span 5’35- 6‘30 [selected]**

F : Which one temperid ? @

T: Americans. @

L: New generation. @

F : A new generation born in this country temperid by the war. It means they @ experienced @

H: Who tempered by war? @

T: The new generation. @

H: A new generation of American born in the war tempered @ by war. @

**time span 6'31-6’55’ delete 24”】**

H: 这个temper 是不是使生气的意思？是不是使生气的意思？使动？被，还是生气了？@

【Discussing with teacher】

**Time span 7:40- 8’35** 【delete 55“】

T: 是个动词。@

F : 锻炼。@

T: Experienced. @

L: 就是experienced. @

H: 就是锻炼？@

F : Exercise. @

H: Train. @

T: Trained by war。唉，也行。Trained by war and ruled by … @

H: 考验，那个词？@

L: Test. @

H: Test? @

L: Test by war. @

H: 这个词，哪个词那么恰当啊？@

L: 锻炼的意思是吧train? @

F : 是系统的训练. @

L: 或者说也不是。@

H: 那你们就都加一起train and test就得了嘛。@

L: Train and test. @

【writing answers, thinking】

**: time span 9‘15“-9’55” [selected]**

L: So what’s the meaning of hard and bitter peace? Or we just change another word. @

T: OK, so difficult. @

L: past. @

T: Peace is very suffery. @

fan: Suffering. @

【reading】

**time span 10’20”- 12’ [selected]**

T: Human rights. @

F : Committed to @ what? @

T: Committed to bargain for human rights. @

F : Human fight for freedom. @

T: “Which”, human right. @

L: “Which” means fight for human rights. @

F : 词性呢？@

H: 词性呢？@

L: Only in English. @

T: Only in English. No more Chinese. @

L: “Which” means fight for our tasks. @

T: What? @

L: “Which” means fight for our rights. @

H: Fight for what? @

L: Human rights. @

H: Human rights. @

F : So we finished. @

H: Human rights. @

F : We are done. We are finished. @

【writing and thinking】

**time span 13’20- 16’14 [selected]**

L: Still this sentence. @

T: Don’t want to. @

F : Yeah. @

T: “Witness” see and … @

L: But no. @

F : “Slow undoing” @

T: slow pace of finish. @

F : The slow progress of the human … @

T: finish the… @ slow progress of achieve, achieving the @ goal. @

F : “Which” means America. @

L: or this nation. @

F : America have always prevote, @ devote to @ human rights. @ And it @ don’t want to … @

T: I I think @ I know “permit” can acknowledge, @ 承认的意思. @

L: What’s the meaning of slow undoing? @

T: Slow undoing 就是@

L: “Undoing” unfinish. @

F : Unfinish. @

H: Cancel is undoing. @

L: “undoing” has another meaning. @

H: “undoing” @ there’s no meaning to do something. Ah, no. @

F : I will not admit the slow … the reason of …@

H: the reason of failure. Should the reason of failure of this @ human rights @

T: “slow undoing” slow, I think progress. @

F : Progress. @

L: It is more close to progress. @

H: This nation is … @

F : That’s another meaning. @

L: We can @ search it in your bar. @

T: Unwilling to witness and see and @ acknowledge @ acknowledge the @

F : Destruction @ undoing means. @

delete【16’14- 16’44 delete 30”】

L: The reason to destroy … @

T: What destroy? @

F : 就是The human rights are being @ how to say 摧毁？@

L: Destroy. @

T: Ruin. @

F : 就是 sowly. @

T: 哦那，the latter @ part? @

**time span 16’45-17’10” [selected]**

F : We cannot bear, we cannot stand with the human rights @ the destr- @ the destroy of this human rights because we @ always @ devote to fighting for human rights. Am I clear? @

T: Slow destroy? @

H: Commit. @

L: No. @

【writing answers】

**time span 18’25”-19‘26“ [selected]**

H: So what’s figure? @

F : Figure? @

L: So we’ve finished question five? @

F : We can still not find. @

T: Ignore it. @

L: Escape it. It’s a little difficult. @ That’s my idea. @

T: OK. @

L: I think @ unwilling to witness or see or allow the @ the just @ American people can’t @ really get the human rights, can really get the human right, which @ the @ the nation had @ has always @ has always, has always promised. @

F : Good 行。@

T: Perfect. @

【writing answers】

**time span 19’56”- 20’04 [selected]**

T: American? @

F : Yeah, reasonable. @

T: I think it’s very … American citizens? @

L: Yeah, Americans. @

【writing answers】

**time span 23’30“-23’40” [selected]**

T: Antithesis. @

L: Antithesis. Some other @ figures? @

delete【24’21-24’56”】

F : “Support” is contrast with “oppose”. Any other? Repetition? @

T: Actually the @ first three @ phrase: hear, bear, meat, @

L: What? @

T: I think antithesis just “support” and “oppose”. @

delete [24’57”-25’15]

T: 有排比吗？@

L: 排比吗？@

H: 排比是这个。Ideas. @

F : No more Chinese. @

**time span 25’16- 26’ [selected]**

H: Ok. Pa-@para-@parallelism. @

T: I think the first three, @ I don’t see a contrast. @

F : Yeah, I don’t see the contrast, @ too. @

T: You mean this changed into this: parallelism? @

L: Parallelism? @

T: Because the first three don’t have a contrast. @

**Time span 26’01- 26’27 [delete]**

L: Where is L (the absent student)? @

F : I don’t know. @

**Appendix 6:** Post-study recording transcript of Control Group 4

*Total voiced recording time*: 18’11”

**time span 1‘- 4‘20“ [Deleted]**

L：叫什么来着？

W： metaphor.

L：什么？

W：metaphor.

L：暗喻？

Y：它有没有明喻啊？

L：嗯？

（Reading）

W：我总感觉还有点对仗。

L：对我也觉得有点对仗的意思，因为它那个end和beginning; 然后 renewal and change.

W：你刚才说的那个明喻、暗喻的问题，是哪个是暗喻?我觉得是暗喻吧。

Y：投票，投票。

C：暗喻是哪里啊，暗喻的？end 和beginning 吗？

L：那个victory of party 吧，如果你们要说的话。

C：其实我个人觉得是那个对仗。看出来吧？

W：有两个都用到了。

C：对仗怎么写？

L：对仗英文怎么写？An-@tithesis, an-@tithe-@antithesis.

(writing answers)

L：party 如果要说是暗喻或排比。

C：排比，也有排比吧。它不是搞两个暗喻不是？

L：排比？

C：对啊。就是symbolizing 什么什么, signifying 什么什么。

L：英文里面两个就是可以排比了是吗？

C：嗯。那排怎么写英文？

B：Para-@dom. Para-@

W：这parallelism.

(writing answers)

Y：我们都写上。

B：我们都写没问题是吧？

C：还要解释吗？

（writing answers）

L：好就这样。大家一致和解了。就这样。一致同意。

W：第三个是什么？

Y：没有就这两个呀。

W：第三个是比喻、暗喻的没有了？

L：大家放弃了。明喻、暗喻还要吗？

Y：没有明喻和暗喻吧。

C：renew 没有其它的吗？

Y：没有明喻，因为没有把什么比作什么。

L：第二段、第二段。第十一行。

[reading, thinking]

**Time span 4’46”- 6’45 [selected]**

G：Forbear 是什么意思呀？

C：Ancestor 就是你的祖先。

L：All men 老师上课说的:人人平等, are created equal.

C：At issue, @ still at issue 就是still do not @ didn’t solved, haven’t been solved 吧？

B：嗯嗯，嗯嗯。

（writing）

L：Forbear 是什么?

C：祖先，就前面那些.

L：Ancestors.

C：yet 就是but 吧？But the @ revolution 这要不要翻？不用翻？But the same revolutionary belief…

L：就是all man are created equal. 老师是这么翻的。(Reading from book) 就是人生而平等。

C：人生而平等。啊！The revolutionary belief, @ such as the @ equality between @ humans @ human being @ and 还有什么？就是一句话吗？

L：然后，等一下，到最后呢？

Y：到 global, @

C：到 global. 啊，那就这句话，就这。a fire 就是 try to achieve 嘛。Try to achieve 达到。主要是still @ still at issue 要翻出来。

L：“At issue” 就是还没得到。就是还没解决。

C：啊，在争论当中。 Is still @ still @ haven’t been solved yet. 就这么写吧？用but 还是yet? But 也行。

L：就用but.

B：while 呢？

C: While? 但是它的重点是前面有一个事情，后面有一个事情，是两个共性的。但是前面就没有共性。

B：Worldwide 是什么意思？

G：世界范围里，大家还没有解决这个问题。

【writing, thinking】

**Time span 9’07” – 13’58”** **[selected]**

G：看不懂。

B：二战算不算bitter peace?

G：嗯，bitter peace,

Y：为什么和平还bitter 呢？

B：战停还bitter?

L：对啊，二战结束就是他享受真正的和平。

W：就是生长于战争然后，

G：是不是来之不易的和平呀？就是经历hard 和 bitter 以后才得到的和平。所以我们要要更加，就是因为为了这种和平要更加注意。

W：那tempered by war 什么意思？

Y：就是在战争中，在战争中那什么不，然后又经历了…

C：temper 是调和、调节。

G：就是，我觉得是不是经历过战争以后我们才，就是慢慢的这种性格就不去再去挑起战争了。就锻炼了。

W：就是在圆滑的世故中磨练之后成长了。

C：那他这也是磨练吗？在战争中磨练。

W：在战争中磨练啊？

C：然后在这么一个痛苦而又艰难的和平中…

L：什么意思？

C：啊，temper 有一个意思是( Reading from dictionary：to become hard and strong by heating it and cooling it.) 通过战争啊磨练。

L：磨练。

C：tempered by war and we being strong after we experienced the war and disciplined by an @

G：disciplined 怎么讲？

C：discipline 应该指的是control，@ control on @ ourselves and … by

W：我觉得是restrain @ restrain 规约。

C：啊restrain, @ constrain?

L：constrain? 规约？

C：就是限制。也就是控制自己，限制自己，自律什么什么。

L：对。关键它这个怎么翻译，什么 hard and bitter peace?

C：hard,

W：应该是来之不易的…

C：by hard 对，我觉得by hard 应该是来之不易的吧 --- difficultly gained, @ difficultly obtained and @ ”bitter” 指的是什么呢？

Y：bitter, @ bitterness

W：我觉得应该是他经历过那些战争所以

G：他知道这个和平夹杂着 … 是痛苦的欢乐吗？

B：嗯对。

C：就像老师说的那个敌人…

Y：是像听义勇军进行曲一样吧。

G：嗯，那个叫什么来着？居安思危吧？

B：怎么感觉不对呢？

C：discipline 就是遵守规则然后有控制，能够控制，控制自己，控制…

G：就是经历过磨难以后我们才知道在和平时代自己要自律的那种吧？因为这个和平我们知道是来之不易的。

L：tempered by war 是什么来着？

C：We are stronger after the …

L：呃，什么经历了战争?

C: bitter 到底怎么写呢？用语言？

L：bitter 英文怎么翻？

G：来之不易的怎么说？

W：不知道。你这个查出来了吗？

C：不bitter 它肯定就是语境，语境就是和他本来的行为没有关系了。

B：bitter

W：是不是因为代价，就是bitter peace

Y：花费的代价太大了，所以大家心理都很苦涩？

L：老师不是讲了两两国，

W：两国打仗然后说那些参，就是…

Y：我觉得这应该是说充满了责任吧？

W：brutal and 血腥的

C：可以这么说，因为它，

W：因为它就是上一节课不是说有个讲了就是谁杀了谁，然后是大家都互相不认识，但是敌人怎么滴，然后无可奈何bitter …

(discussing with teacher)

**Time span 14’27- 14’57 [selected]**

Y：which, 下面一个问题是which.

W：赶紧说中文，不然没机会了。

B：妈呀，这句话也被记录进去了。

Y：21行的which 赶紧找一下，它指的是什么?

（reading, thinking）

**Time span 15’48- 17’13 [selected]**

W：哦，这句话好长啊！

G：Human rights? 还是ancient heritage?

C：那这个 hard 怎么翻译呢？就是disciplined by a hard and bitter @ hard?

G：Hard 不用翻了。就是hard。就直接写hard，把bitter 翻译一下就行。

C：Bitter 老师说就是hard 的意思。

L：Hard, uneasy.

C：哦对，我想起来了，你们继续。

L：啊，这句话太长了。

C：哎在哪个地方呢这个which?

L：21行。第一个。

G：我觉得是ancient heritage.

C：We can just speak English.

**Time span 17’33”- 20‘14“ [selected]**

B：按正常语序是：unwilling to witness @ the @

L：就是后面那个which 是不是一样的？

W：是一样的。哦不是一样的。

C：这有两个which 啊。

Y：哪有两个which? 一个which.

G：后面还有一个which. 21页。

C：它指的是最后一个which.

L：嗯？不是第一个？

W：最后一个committed to which

Y：nation 吧这个。

C：啊，这到底是哪里啊?

Y：committed to 吧？

L：对。

Y：committed to 什么，坚定的什么？

G：就先看前面的

W：整个都是一句话。Which 指的是human rights.

C：我是觉得是human rights. @ They committed to achieving the @ human rights. 但是这前面没有用到这个词。[reading from book and then said] They are committed to the human rights, but now the situation is that @ the @ process to get the human rights is the slow undoing.

W：slow doing.

C：Very slow but also didn’t achieve the human rights. Undoing I think it means they didn’t achieve, they didn’t gain the human rights.@ So we committed to @ must refers to the human rights @ they want to gain.

W：Sure. Human rights.

C：Any other opinions? If didn’t, we will said that.

G：Oh, my God!

C：The sentence means we want to get the human rights, but we haven’t yet @ done it. So I think the “which” refers to.

L：Next question. Paraphrase this sentence.

**Time span 20’44- 21:10 [selected]**

L：Our nations are @ always committed to @ committed to @ committed to @ the human rights to @

C：Just we don’t want to @ we don’t’ want to see @ and

**Time span 21’27- 22’45**   **[selected]**

C：We just have two minutes.@ Permit means allow it @ happen. @ We didn’t allow it happen. We don’t want to see and we don’t want it happen that @ the slow

W：We could add a to to human rights.

C：In a very slow

W： That is.

C：Ah, Ok.

G：The human rights that the nation had committed to us @ can not be achieved.

L：Yes, yes.

G：好下一个。Next question. @ 是排比吗？Parallel.

Y：在哪呢？

**Time span 23’10- 24’10 [selected]**

G：Repetition. 哦不对。

B：Parallism.

G：Parallelism and @ alliteration.

Y：头韵法。头韵法在哪？

L：P-P

B：pay any price, bear any burden.

Y：啊！头韵法吗？

L：前面有两个。

Y：哪两个？

L：Pay and price, bear and burden.

【writing answers】

**Time span 25’19-26’20 [selected]**

Y：其实不能说一下，还有一句话不懂呢。这句and yet the same revolutionary belief … 她等会儿提问我不会。

C: 哪个？哪个地方不会？

Y：我不知道这句话是什么意思。

C：这句话的意思是：即使，即使 ……

L：即使我们的祖先为了，为了这个东西而奋斗，但是这个问题还是没有解决。为了人人平等而奋斗，但是这个问题还是没有解决。

C：即使这些革命性的理念比如说追求人人平等，我们祖先已经一直在追求，一直在努力去实现了，但是还是没有实现。而且必须还是要在全世界的范围内得到解决。
